# Low cigarette consumption and risk of coronary heart disease and stroke: meta-analysis of 141 cohort studies in 55 study reports

**DOI:** 10.1136/bmj.j5855

**Published:** 2018-01-24

**Authors:** Allan Hackshaw, Joan K Morris, Sadie Boniface, Jin-Ling Tang, Dušan Milenković

**Affiliations:** 1 Cancer Research UK and UCL Cancer Trials Centre, University College London, London W1T 4TJ, UK; 2 Wolfson Institute of Preventive Medicine, Queen Mary, University of London, London, UK; 3 Addictions Department, Kings College London, London, UK; 4 JC School of Public Health and Primary Care, Chinese University of Hong Kong, Hong Kong; 5 Meta Research, Evidera, London, UK (formerly Cancer Research UK and UCL Cancer Trials Centre)

## Abstract

**Objective:**

To use the relation between cigarette consumption and cardiovascular disease to quantify the risk of coronary heart disease and stroke for light smoking (one to five cigarettes/day).

**Design:**

Systematic review and meta-analysis.

**Data sources:**

Medline 1946 to May 2015, with manual searches of references.

**Eligibility criteria for selecting studies:**

Prospective cohort studies with at least 50 events, reporting hazard ratios or relative risks (both hereafter referred to as relative risk) compared with never smokers or age specific incidence in relation to risk of coronary heart disease or stroke.

**Data extraction/synthesis:**

MOOSE guidelines were followed. For each study, the relative risk was estimated for smoking one, five, or 20 cigarettes per day by using regression modelling between risk and cigarette consumption. Relative risks were adjusted for at least age and often additional confounders. The main measure was the excess relative risk for smoking one cigarette per day (RR_1_per_day_−1) expressed as a proportion of that for smoking 20 cigarettes per day (RR_20_per_day_−1), expected to be about 5% assuming a linear relation between risk and consumption (as seen with lung cancer). The relative risks for one, five, and 20 cigarettes per day were also pooled across all studies in a random effects meta-analysis. Separate analyses were done for each combination of sex and disorder.

**Results:**

The meta-analysis included 55 publications containing 141 cohort studies. Among men, the pooled relative risk for coronary heart disease was 1.48 for smoking one cigarette per day and 2.04 for 20 cigarettes per day, using all studies, but 1.74 and 2.27 among studies in which the relative risk had been adjusted for multiple confounders. Among women, the pooled relative risks were 1.57 and 2.84 for one and 20 cigarettes per day (or 2.19 and 3.95 using relative risks adjusted for multiple factors). Men who smoked one cigarette per day had 46% of the excess relative risk for smoking 20 cigarettes per day (53% using relative risks adjusted for multiple factors), and women had 31% of the excess risk (38% using relative risks adjusted for multiple factors). For stroke, the pooled relative risks for men were 1.25 and 1.64 for smoking one or 20 cigarettes per day (1.30 and 1.56 using relative risks adjusted for multiple factors). In women, the pooled relative risks were 1.31 and 2.16 for smoking one or 20 cigarettes per day (1.46 and 2.42 using relative risks adjusted for multiple factors). The excess risk for stroke associated with one cigarette per day (in relation to 20 cigarettes per day) was 41% for men and 34% for women (or 64% and 36% using relative risks adjusted for multiple factors). Relative risks were generally higher among women than men.

**Conclusions:**

Smoking only about one cigarette per day carries a risk of developing coronary heart disease and stroke much greater than expected: around half that for people who smoke 20 per day. No safe level of smoking exists for cardiovascular disease. Smokers should aim to quit instead of cutting down to significantly reduce their risk of these two common major disorders.

## Introduction

Around one billion adults worldwide smoke,^1^ with high prevalence in developing countries, where 49% of men and 11% of women use tobacco.^2^ Although the prevalence of current smokers has decreased over time in several countries, the global absolute number of smokers has increased owing to population growth.^3^ Policies have successfully encouraged people to quit, using aids such as nicotine replacement therapy and electronic cigarettes (e-cigarettes).^4^

In the Health Survey for England (2013 and 2014), 26% of current smokers reported that they wanted to cut consumption down but were not trying to stop, and 40-41% said that they smoked less than in the previous year.^5^ The percentage of smokers who consume one to five cigarettes per day has steadily risen (from 18.2% to 23.6% between 2009 and 2014^5^), with a similar pattern in the US, where the proportion of smokers who consume less than 10 cigarettes per day increased from 16% to 27% between 2005 and 2014.^6^ A recent Cochrane review discussed the evidence for ways of helping smokers who wish to reduce their consumption.^7^

Smoking few cigarettes is generally believed to be relatively safe, as has been incorrectly assumed for light/low nicotine cigarettes.^8^ Among 24 658 US adolescents, 10% thought that light smoking was not harmful, and only 35% of light smokers considered their habits to be associated with “a lot of harm.”^9^ Reducing consumption might be expected to reduce harm in a proportionate way—that is, that smoking one instead of 20 cigarettes per day has about one twentieth (5%) of the risk. This seems to be the case for lung cancer, for which the large American Cancer Society Prevention Study II showed an approximately linear relation between risk of lung cancer and number of cigarettes smoked per day, but the dose-response for cardiovascular disease is steep at low consumption and then levels off,^10^ consistent with the shape reported previously.^11^

In a seminal systematic review of second-hand smoke and coronary heart disease among never smokers published in the *BMJ* 20 years ago, Law and colleagues drew attention to the 1.30 risk ratio being relatively large compared with the 2-3 typically seen in studies of active smokers.^12^ Their conclusions on second-hand smoke were supported by a meta-analysis of active cigarette smoking and risk of coronary heart disease from five cohort studies, in which the modelled relative risk for smoking one cigarette per day (1.39) was consistent with that for exposure to second-hand smoke.

Although the non-linear relation between coronary heart disease and low cigarette consumption has been reported before (individual studies, as well as official reports from the US Surgeon General), it still is still not commonly known by the general public or health professionals, particularly those not involved in tobacco and health. We thus aimed to extend the previous work on coronary heart disease,^12^ by using a systematic review to provide a major body of evidence. We also aimed to show that a similar non-linear relation exists between stroke and low cigarette consumption.

## Methods

### Data sources and searches

We did a systematic literature review of English language articles published between 1946 and May 2015 in Medline (MOOSE guidelines^13^) that reported the association between cigarette consumption and coronary heart disease and stroke. Supplementary figure A shows the search terms and flowchart: 13 861 abstracts were reviewed (by DM and SB), and any selected for consideration had their reference list manually checked for additional studies. Several study reports were based on combining data from at least two separately conducted cohort studies.

### Study selection and data extraction

We included prospective cohorts with at least 50 cardiovascular disease events (mortality, morbidity, or both) to minimise the potential for reporting bias, in which large but unreliable effects might be seen in small studies. Reports had to give hazard ratios from a Cox proportional hazards regression or relative risks based on incidence/mortality, which must have been adjusted by at least age, or incidence reported in age groups. Results had to be available in at least three smoking categories, not including the reference group of never smokers. The populations of the cohorts had to be generally healthy; we excluded studies based only on people at high risk (for example, taking drugs for cardiac related disorders). Results had to be given separately for men and women, or, if they were based on both combined, the hazard ratios must be adjusted for age and sex. We excluded six studies spuriously showing that the hazard ratio or relative risk decreased with increasing consumption (justification in supplementary figure A). Study characteristics extracted were country, time period, sex, smoking categories, incidence, hazard ratio or relative risk, number of participants, number of events, and confounding factors adjusted for. In the few instances in which only age adjusted incidence/mortality results were available, we calculated the relative risk in each smoking category. Most studies reported hazard ratios, and we always used hazard ratios adjusted for multiple factors when provided (supplementary table A); 30 of the 55 publications made allowance for multiple (at least two) factors in addition to age and sex when providing hazard ratios. We extracted hazard ratios and relative risks separately for coronary heart disease, stroke, or cardiovascular disease (coronary heart disease and stroke combined).

### Statistical methods

Hereafter, we refer to hazard ratio or relative risk as relative risk (consistent with many studies included). Instead of modelling risk with consumption for each study (which is non-linear), we modelled the logarithm of risk, using similar methods as before.^12^ ^14^ This involved fitting a log-linear variance weighted regression model between incidence or relative risk and cigarette consumption (using all reported smoking categories in the publications). Although this approach makes the relation more linear (when examined on a log scale), it might still underestimate the increase in risk at very low consumption levels.

We obtained a regression model for each study report separately (Stata software). For consumption, we used the midpoint of the reported number of cigarettes per day—for example, three cigarettes per day if the category was one to five cigarettes per day—which we then adjusted for carboxyhaemoglobin and cotinine because this allows for lower inhalation with increasing cigarette consumption as previously established.^14^ For studies that reported relative risks adjusted for age (or for additional factors), the model contained the logarithm of the relative risk (dependent variable) and consumption (independent variable) using only the midpoint of the cigarettes per day categories. For studies that reported incidence in each age category, we fitted log-linear model that contained incidence (dependent variable) and consumption (independent variable) with age as a covariate (median age in each age category), and we estimated the relative risk by using an interaction term between age and consumption. This provided estimates in each age category (45, 55, and 65 years) because the risk of cardiovascular disease changes with age.^15^ The reference value of 1.0 (never smokers) was not included in the regression to avoid forcing the model through the origin and unduly affecting the dose-response relation (also because we were ultimately interested only in comparing between high and low consumption). We used the standard error of the logarithm of the relative risk, or the number of events if the standard error was unavailable, as weights in the regression; if both were unavailable, we did an unweighted log-linear regression for the study. The reference group was lifelong never smokers, although in seven reports it was unclear whether former smokers might have been included.

The main quantitative measure was the percentage change in risk (excess relative risk) associated with smoking one (or five) cigarette(s) per day, expressed as a proportion of the percentage change for smoking 20 cigarettes per day. For example, if the relative risks were 1.4 and 1.9 for smoking one and 20 cigarettes per day, respectively, the proportion of excess relative risk associated with one cigarette per day is 44%: (1.4−1)/(1.9−1)×100. One or five cigarettes per day reflect typical levels of low consumption. We did three different types of analyses, to check for consistency. Firstly, from each regression analysis for each study, we used the model to estimate the relative risk for smoking one cigarette per day compared with never smokers, and also for smoking five and 20 cigarettes per day. We then calculated the excess relative risks for one and five cigarettes per day (compared with 20) and took the median value of each of these across studies. We did multiple separate analyses according to combinations of sex and disease type (“within study” analyses). Secondly, we obtained a single regression model across all studies (again done separately for each combination of sex and disorder) by using the individual cigarettes per day values and reported relative risk estimates (log scale) in a random effects meta-regression (SAS Proc Mixed). We then used the pooled coefficients to estimate the relative risk for one, five, and 20 cigarettes per day (another “within study” analysis). We also used these regressions to examine whether a quadratic trend might be better than a linear trend but found no evidence of this (the quadratic coefficients were negligible and not statistically significant). Thirdly, from the log-linear regression model in each study, we estimated the relative risk for smoking one cigarette per day and then combined these across studies in a random effects meta-analysis, fitted separately for each disease group and sex, using RevMan; we repeated this for smoking five and 20 cigarettes per day. These results (and corresponding diagrams) indicate the variability in relative risk in each smoking group across studies, but they do not directly reflect the within study correlation between risk and consumption (as in the first and second analyses above).

The results are examined in relation to assuming that smoking one cigarette per day is associated with about 5% of the excess relative risk when smoking 20 cigarettes per day. Our regressions used a logarithmic scale, so smoking one cigarette per day would actually have 3.5% or 5.5% of the excess risk if the relative risk for 20 cigarettes per day was 2.0 or 3.0, respectively, values typically seen in the studies (log(relative risk for 20 cigarettes per day)=20×log(relative risk for one cigarette per day)).

### Patient involvement

No patients were involved in setting the research question or the outcome measures, nor were they involved in developing plans for design or implementation of the study. No patients were asked to advise on interpretation or writing up of results. There are no plans to disseminate the results of the research to study participants or the relevant patient community. We did not evaluated whether the studies included in the meta-analysis had any patient involvement.

## Results

The meta-analyses were based on 141 separately conducted cohort studies contained in 55 study reports (several involved the pooling of multiple studies),^16^ ^17^ ^18^ ^19^ ^20^ ^21^ ^22^ ^23^ ^24^ ^25^ ^26^ ^27^ ^28^ ^29^ ^30^ ^31^ ^32^ ^33^ ^34^ ^35^ ^36^ ^37^ ^38^ ^39^ ^40^ ^41^ ^42^ ^43^ ^44^ ^45^ ^46^ ^47^ ^48^ ^49^ ^50^ ^51^ ^52^ ^53^ ^54^ ^55^ ^56^ ^57^ ^58^ ^59^ ^60^ ^61^ ^62^ ^63^ ^64^ ^65^ ^66^ ^67^ ^68^ ^69^ ^70^ ^70^ and two other study reports are referred to later on.^71^ ^72^ Table 1 shows all summary results.

**Table 1:** Relative risk of cardiovascular disease for smoking one, five, or 20 cigarettes per day (CPD): summary results from meta-analyses

Cohort	No of study reports	Approximate No of participants	Approximate No of events	Pooled relative risk (95% CI) for smoking (compared with never smokers)*				Excess relative risk, as % of that for 20 CPD†	
				1 CPD	5 CPD	20 CPD		1 CPD	5 CPD
**Coronary heart disease**									
Men	26	2.31 million	57 152	1.48 (1.30 to 1.69); (1.45)‡	1.58 (1.39 to 1.80); (1.56)‡	2.04 (1.86 to 2.24); (2.06)‡		46; (46)*; (42)‡	57; (56)*; (53)‡
Women	18	2.34 million	29 870	1.57 (1.29 to 1.91); (1.59)‡	1.76 (1.46 to 2.13); (1.79)‡	2.84 (2.21 to 3.64); (2.81)‡		31; (31)*; (33)‡	43; (41)*; (44)
Combined	5	1.01 million	15 153	1.65 (1.53 to 1.78); (1.67)‡	1.72 (1.62 to 1.83); (1.81)‡	2.34 (1.96 to 2.79); (2.44)‡		53; (49)*; (47)‡	61; (54)*; (56)‡
Men aged:									
45 years	8	938 000	27 697	1.65 (1.26 to 2.16)	1.81 (1.40 to 2.33)	2.72 (2.16 to 3.43)		35	46
55 years	8			1.41 (1.17 to 1.70)	1.51 (1.27 to 1.80)	2.03 (1.74 to 2.36)		33	44
65 years	8			1.17 (0.96 to 1.43)	1.24 (1.03 to 1.48)	1.49 (1.28 to 1.74)		20	36
Women aged:									
45 years	3	555 000	14 665	1.26 (0.98 to 1.62)	1.34 (0.92 to 1.96)	2.19 (1.11 to 4.32)		11	26
55 years	3			1.21 (1.05 to 1.39)	1.26 (0.98 to 1.62)	1.77 (1.00 to 3.11)		15	28
65 years	3			1.15 (1.06 to 1.25)	1.24 (1.11 to 1.40)	1.47 (0.94 to 2.29)		36	45
**Stroke**									
Men	17	3.40 million	71 173	1.25 (1.13 to 1.38); (1.37)‡	1.30 (1.18 to 1.43); (1.42)‡	1.64 (1.48 to 1.82); (1.62)‡		41; (39)*; (60)‡	52; (47)*; (68)‡
Women	10	3.59 million	60 520	1.31 (1.13 to 1.52); (1.35)‡	1.44 (1.22 to 1.70); (1.48)‡	2.16 (1.69 to 2.75); (2.13)‡		34; (27)*; (31)‡	44; (38); (42)‡
Combined	2	228 000	2874	1.52 (1.10 to 2.10); (1.56)‡	1.63 (1.19 to 2.21); (1.65)‡	1.90 (1.54 to 2.35); (2.03)‡		58; (58)*; (54)‡	66; (70)*; (63)‡
Men aged:									
45 years	2	315 000	4456	1.41 (1.03 to 1.94)	1.62 (1.26 to 2.09)	2.89 (2.31 to 3.62)		22	35
55 years	2			1.27 (1.02 to 1.57)	1.39 (1.09 to 1.75)	2.01 (1.46 to 2.76)		25	43
65 years	2			1.18 (0.90 to 1.54)	1.21 (0.89 to 1.64)	1.44 (0.96 to 2.15)		15	30
Women aged:									
45 years	1	534 000	5512	1.40 (0.93 to 2.11)	1.60 (1.14 to 2.24)	2.64 (2.20 to 3.17)		24	37
55 years	1			1.25 (0.95 to 1.64)	1.41 (1.13 to 1.76)	2.22 (1.97 to 2.51)		20	34
65 years	1			1.12 (0.85 to 1.47)	1.25 (1.00 to 1.56)	1.87 (1.66 to 2.11)		14	29
**Cardiovascular disease (coronary heart disease and stroke not reported separately)**									
Men	7	111 000	3480	1.45 (1.00 to 2.11); (1.61)‡	1.59 (1.11 to 2.26); (1.70)‡	2.19 (1.56 to 3.09); (2.10)‡		20; (38)*; (55)‡	34; (50)*; (64)‡
Women	1	153 000	2768	1.65 (1.13 to 2.40)	1.74 (1.30 to 2.34)	2.16 (1.69 to 2.76)		56; (56)*	64; (64)*
Combined	4	1.00 million	36 525	1.63 (1.53 to 1.73); (1.64)‡	1.71 (1.63 to 1.80); (1.75)‡	2.27 (1.96 to 2.62); (2.25)‡		50; (50); (51)‡	60; (56)*; (60)‡

* From combining relative risk for one CPD across all studies (and again, separately, for five and 20 CPD). Although they do not reflect within study correlations, in most cases they are close to those obtained from fig 2 and also meta-regressions (both of which are based on within study analyses).

† From within study analyses (fig 2); they represent median values across studies.

‡ Estimates obtained from single meta-regression model across all studies (for men and women separately and for each disorder).

### Coronary heart disease

**Fig 1:**
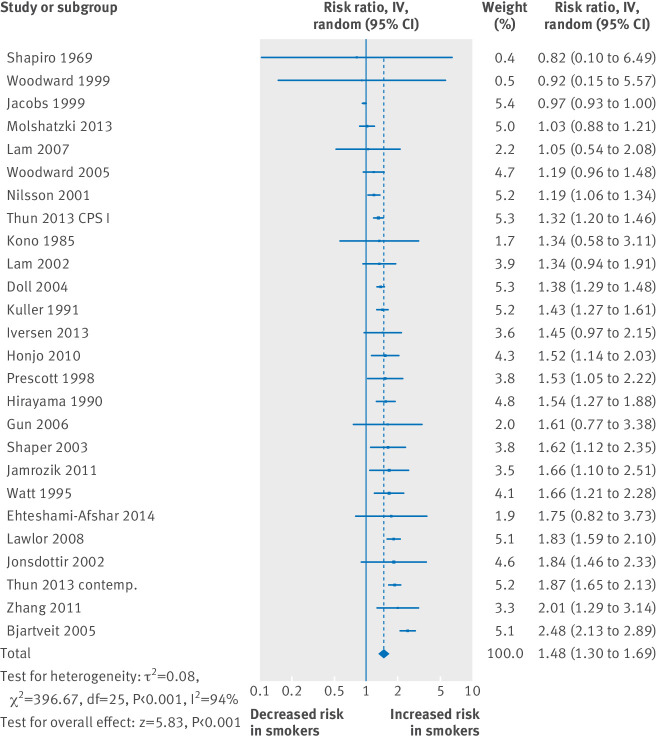
Relative risk for coronary heart disease for men smoking one cigarette per day. IV=inverse variance. Studies are in reference numbers 16-70. Excluding five studies that used relative risks instead of hazard ratios increased pooled relative risk (to 1.53)

**Fig 2:**
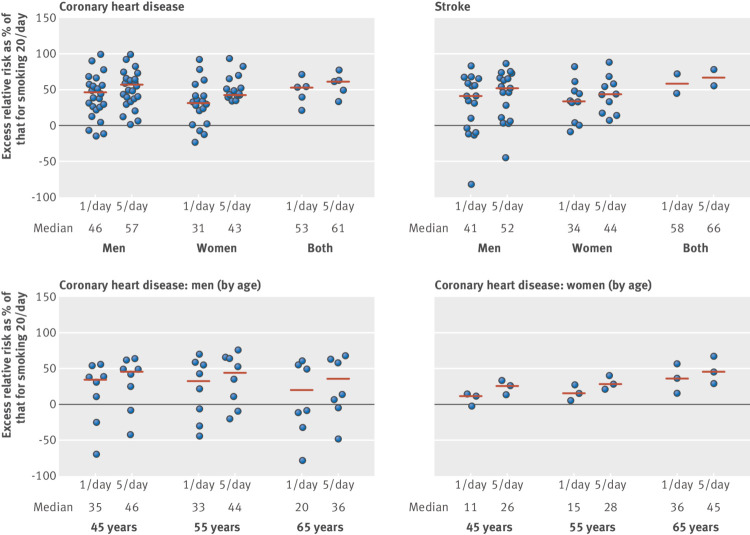
Distribution of excess relative risk for smoking one or five cigarettes per day, each in relation to smoking 20 per day, using within study results (horizontal dashes show median). For example, in Lawlor et al (2008),^48^ estimated relative risk for coronary heart disease (CHD) was 1.83 or 2.63 for those smoking one or 20 per day, respectively (from regression analysis of this study). Proportion of excess relative risk associated with one cigarette per day is therefore 51%: (1.83−1)/(2.63−1), which is plotted. (A negative value is when relative risk for one (or five) per day is <1.0.) For CHD in men, one study (Wen et al 2004)^66^ reported decreasing relative risks for increasing consumption for ≥65 age group, which appears as excess relative risk percentage of >100% (for completeness these are kept in, but do not affect median value)

The pooled relative risk from 26 study reports was 1.48 (95% confidence interval 1.30 to 1.69) for men who smoked, on average, one cigarette per day and 1.58 (1.39 to 1.80) for those who smoked five cigarettes per day; the relative risk for smoking 20 cigarettes per day was 2.04 (1.86 to 2.24) (fig 1; supplementary figure B). (Excluding three studies that might have included former smokers in the reference group increased the relative risks for one and 20 cigarettes per day to 1.53 and 2.09, as expected.) Figure 2 shows the distribution of the excess relative risks; most had values of at least 25%. Using within study comparisons, smoking one cigarette per day had 46% (interquartile range 24-56%) of the excess relative risk for that when smoking 20 cigarettes per day, and the corresponding estimate for five cigarettes per day was 57% (36-64%).

**Fig 3:**
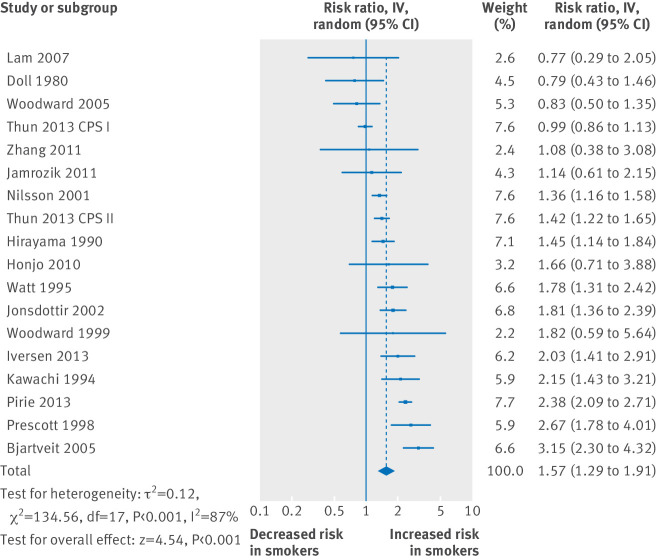
Relative risk for coronary heart disease for women smoking one cigarette per day. IV=inverse variance. Studies are in reference numbers 16-70. Excluding two studies that used relative risks instead of hazard ratios slightly increased pooled relative risks (to 1.63).

The 18 reports of women showed that one cigarette per day had 31% (interquartile range 2-46%) of the excess risk of 20 cigarettes per day (pooled relative risks 1.57 *v* 2.84), and smoking five cigarettes per day had 43% (14-55%) the excess risk (relative risk 1.76) (fig 3; supplementary figure C. (Excluding one study that might have included former smokers in the reference group increased the relative risks for one and 20 cigarettes per day to 1.63 and 2.87.)

All of these estimates were similar to those obtained from the meta-regression (using a single model across studies) (table 1). Also, the relative risk estimates for one, five, and 20 cigarettes per day were mostly similar when produced by pooling these separately across studies (not within study analysis) to those from the meta-regressions (within study analysis).

There was a suggestion that the relative risks at low consumption might be higher for women than for men (1.57 *v* 1.48 for one cigarette per day; 1.76 *v* 1.58 for five cigarettes per day), consistent with a higher risk of coronary heart disease in women reported by others.^73^ A comparison between sexes could also be examined directly within the same study cohort, where a higher relative risk was seen, without modelling: Hirayama et al (relative risk 1.61 for women versus 1.50 for men, for smoking one to four cigarettes per day),^29^ Nilsson et al (1.47 *v* 1.24, for smoking one to seven cigarettes per day),^53^ Prescott et al (2.14 *v* 1.03, for smoking three to five cigarettes per day),^72^ and Bjartveit et al (2.94 *v* 2.74, for smoking one to four cigarettes per day).^17^

Supplementary figure D shows the forest plots for the age and sex adjusted relative risks in five studies for which results were not reported separately by sex: consuming one or five cigarettes per day had 53% or 61% of the excess risk, compared with 20 cigarettes per day (table 1). Supplementary figures E and F are the forest plots for coronary heart disease and smoking consumption in men and women separately for people aged 45, 55, and 65 years. The individual relative risks among men reflect the decreasing strength of association between coronary heart disease and smoking as people get older. The excess risk for smoking one cigarette per day expressed as a percentage of that for 20 cigarettes per day remained high throughout (fig 2): 35%, 33%, and 20% for a man aged 45, 55, and 65 years, respectively; the corresponding figures for women were 11%, 15%, and 36% (in which the older age group seems to have a larger estimate, but there were only three studies here). Table 1 shows the results for five cigarettes per day.

All estimates (men, women, and both together) are much higher than the expected 5% had a linear or log-linear relation existed between consumption and risk.

### Stroke

**Fig 4:**
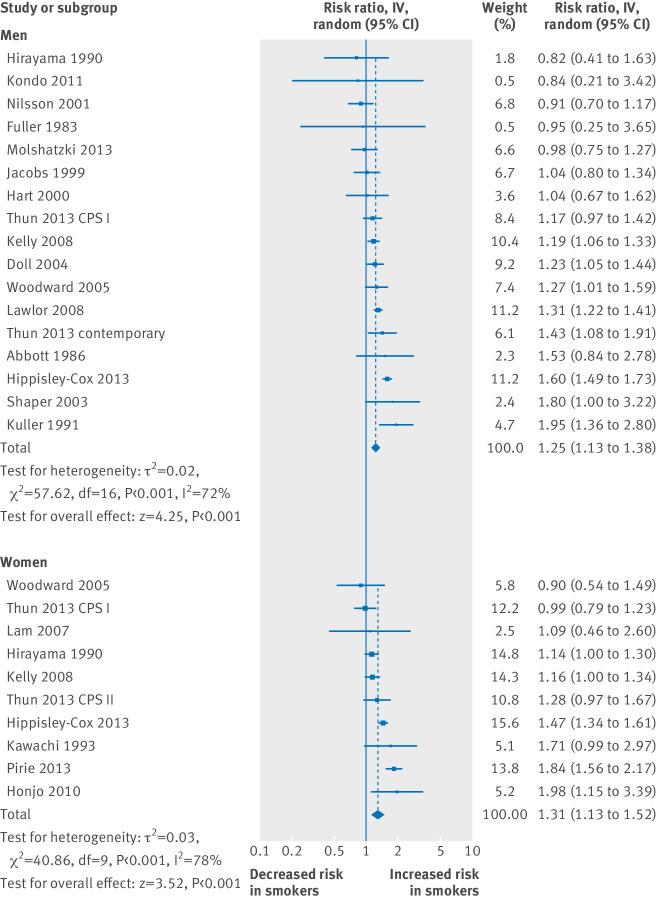
Relative risk for stroke for smoking one cigarette per day in men (top) and women (bottom). Studies are in reference numbers 16-70. IV=inverse variance. Excluding four studies in men and one study in women that used relative risks instead of hazard ratios slightly increased pooled relative risks to 1.28 for men and 1.34 for women

Figure 4 and supplementary figures G and H show the relative risks for stroke. Among men who smoked one cigarette per day, the relative risk was 1.25 (1.13 to 1.38); for women, it was 1.31 (1.13 to 1.52). The corresponding estimates for smoking 20 cigarettes per day were 1.64 (1.48 to 1.82) and 2.16 (1.69 to 2.75). These are again consistent with a slightly larger effect of smoking in women at the lowest smoking levels but more so at higher consumption, compared with men (1.44 *v* 1.30 for five cigarettes per day; 2.16 *v* 1.64 for 20 cigarettes per day), as seen elsewhere.^73^

From the within study analyses (fig 2), the distribution of excess relative risks again showed that most exceeded 25%. Smoking one cigarette per day had an estimated 41% (interquartile range −7-62%) of the excess relative risk of men who smoked 20 cigarettes per day (from 17 studies), and the corresponding figure for five cigarettes per day was 52% (9-70%). These were similar to the findings in women (10 studies), in whom one cigarette per day had 34% (3-51%) of the excess risk of 20 cigarettes per day and five cigarettes per day had 44% (16-60%).

Supplementary figure I shows the forest plots for the age and sex adjusted relative risks. Supplementary figure J shows the forest plots for stroke and cigarette consumption in men according to age. The excess risk for smoking one cigarette per day expressed as a percentage of that for 20 cigarettes per day was 22%, 25%, and 15% for a man aged 45, 55, and 65 years (two studies); the corresponding figures for women were 24%, 20%, and 14% (although these were based on only one study).

As with coronary heart disease, all estimates for stroke (men, women, and both together) were much higher than the 5% value expected with a linear or log-linear relation.

### All cardiovascular disease

Supplementary figures K and L are forest plots for cardiovascular disease (coronary heart disease and stroke reported together), showing adjusted relative risks in men or women. Again, results were consistent with those seen for each disorder separately.

### Heterogeneity and bias

The heterogeneity seen in some meta-analyses is largely due to statistically significant relative risk estimates that differ from each other, and several reasons for this may exist (for example, with or without adjustment for multiple confounders). In figure 1, 15 estimates for one cigarette per day were each statistically significant, ranging between 1.19 and 2.48. However, even the lowest relative risk of 1.19 is a significant increase in risk of coronary heart disease (representing 25% of the excess risk compared with its corresponding estimate for 20 cigarettes per day: relative risk=1.77).

We explored the possibility that some heavy smokers reduced to light smoking during the course of the study, which in turn might substantially reduce the relative risks in the high consumption categories, moving them closer to that for light smokers, when using baseline consumption to produce relative risks. This could overestimate the excess relative risk for one to five cigarettes per day when compared with that for 20 cigarettes per day. Such changes in smoking habits are expected to have largely occurred in the later years, so we examined only studies that had follow-up to 1995, to see whether the relative risks were much higher than those based on all studies. This was not the case. The pooled relative risks for coronary heart disease associated with smoking 20 cigarettes per day were 1.8 (1.6 to 2.0) for men and 2.5 (2.0 to 3.1) for women, a modest reduction compared with 2.0 and 2.8 from all studies in table 1. Also, we found no evidence of a negative trend between size of relative risk for smoking 20 cigarettes per day and last calendar year of follow-up (which might suggest many heavy smokers cutting down, and whether this increases over time): Spearman’s correlations were positive: 0.30 (P=0.15) for men and 0.33 (P=0.20) for women (coronary heart disease studies).

Three large studies (from different countries: Denmark, Norway, and South Korea) specifically examined the effect of reduced smoking on risk of cardiovascular disease. In one study (19 423 adults), only 7.2% of “heavy” smokers (at least 15 cigarettes per day) reduced their consumption by at least 50% but continued to smoke when assessed five to 10 years after baseline (verified by carbon monoxide or cotinine concentrations). There was no clear risk reduction for coronary heart disease compared with continuing heavy smokers after 14 years’ follow-up (adjusted relative risk 1.06), in contrast to a relative risk of 0.67 for quitters.^74^ However, a large reduction in risk of lung cancer was seen in the group who reduced consumption (relative risk 0.44).^75^ In the second study (51 210 adults), 4.2% of heavy smokers (at least 15 cigarettes per day) reduced their consumption by at least 50% but continued to smoke when recorded three to 13 years after baseline. The adjusted relative risk for cardiovascular disease after 21 years’ follow-up was 1.02 (compared with continuing heavy smokers), unlike the benefit seen in quitters (relative risk 0.46) or the positive effect on risk of lung cancer in those who reduced (relative risk 0.66).^76^ In the third study (475 734 adults), 5.2% of heavy smokers (at least 20 cigarettes per day) reduced to less than 10 cigarettes per day two years later, with little risk reduction after nine years’ follow-up (adjusted relative risk 0.85 for stroke and 0.92 for coronary heart disease, compared with continuing heavy smokers), in contrast to the beneficial effect in quitters (relative risk 0.70 for stroke and 0.43 for coronary heart disease)^77^ and the effect on lung cancer in those who reduced (relative risk 0.66).^78^ These studies indicate that a substantial bias is unlikely to be produced by heavy smokers cutting down, because only a small proportion did so, and that those who reduced consumption did not seem to have much benefit in terms of cardiovascular disease risk.

### Model reliability

We checked the reliability of the regression models by comparing the estimated relative risks for smoking one, five, and 20 cigarettes per day with those seen in several individual studies that reported results specifically for low consumption (one to seven cigarettes per day). Our modelled estimates were close to those observed (supplementary table B). A high excess relative risk (in comparison with 20 cigarettes per day) was seen in 17 of 20 estimates (median 57% all estimates; 49% for coronary heart disease and 62% for stroke, comparable to those from the meta-analyses). Supplementary figure M shows examples of individual studies of coronary heart disease or stroke, plotting the observed (reported) relative risks with the ones we estimated using the log-linear model; the fit was generally good.

### Confounding

We explored the influence of confounding factors by doing meta-analyses according to whether studies made allowance for three or more factors (which in addition to age included cholesterol for studies of coronary heart disease and cholesterol or blood pressure for studies of stroke) (table 2). One study did not adjust for either cholesterol or blood pressure but made allowance for multiple other confounders, so we also included it with the “adjusted” group.^55^ Additional factors often included body mass index, education, history of diabetes, and physical activity (see supplementary table A).

**Table 2:** Meta-analyses according to whether studies made allowance for multiple confounding factors

Cohort and analysis*	No of studies	From pooling results for 1 and 20 CPD separately across studies				From meta-regressions (uses within study analyses)		
		RR (95% CI) for 1 CPD	RR (95% CI) for 20 CPD	Excess RR (%)†		RR for 1 CPD	RR for 20 CPD	Excess RR (%)†
**Coronary heart disease**								
Men:								
Adjusted	11	1.74 (1.50 to 2.03)	2.27 (1.90 to 2.72)	58		1.65	2.22	53 (54)
Unadjusted	15	1.36 (1.18 to 1.56)	1.89 (1.71 to 2.08)	40		1.33	1.91	36 (38)
Women:								
Adjusted	9	2.19 (1.84 to 2.61)	3.95 (3.34 to 4.67)	40		2.12	3.98	38 (34)
Unadjusted	9	1.26 (1.07 to 1.49)	2.11 (1.91 to 2.34)	23		1.28	2.12	25 (23)
**Stroke**								
Men:								
Adjusted	6	1.30 (1.11 to 1.53)	1.56 (1.31 to 1.86)	54		1.35	1.55	64 (62)
Unadjusted	11	1.20 (1.07 to 1.35)	1.68 (1.45 to 1.95)	29		1.26	1.68	38 (34)
Women:								
Adjusted	5	1.46 (1.20 to 1.78)	2.42 (1.67 to 3.52)	32		1.50	2.39	36 (33)
Unadjusted	5	1.15 (0.98 to 1.35)	1.94 (1.44 to 2.61)	16		1.14	1.91	15 (34)

CPD=cigarettes per day; RR=relative risk compared with never smokers.

* Adjusted includes only studies that reported RRs after allowance for ≥3 multiple confounders (which includes cholesterol for coronary heart disease studies and cholesterol or blood pressure for stroke studies), plus another study that made multi-factor adjustments.^59^ Unadjusted includes all other studies (although all allowed for age and occasionally one more factor).

† Percentage excess RR for smoking 1 CPD as percentage of that for 20 CPD. Numbers in parentheses are from same type of analyses as in fig 2 (that is, median value from within study comparisons).

Among men, 11 studies of coronary heart disease had multivariable adjusted relative risks,^17^ ^22^ ^32^ ^36^ ^46^ ^47^ ^48^ ^58^ ^60^ ^67^ ^70^ and the pooled relative risks were 1.74 and 2.27 for smoking one and 20 cigarettes per day (table 2). From the meta-regressions, one cigarette per day has 53% of the excess relative risk of 20 cigarettes per day. These adjusted relative risks were higher than those obtained from the 15 other studies that did not allow for multiple confounders: 1.36 and 1.89 for one and 20 cigarettes per day, and the excess relative risk for one cigarette per day is 36% (lower than the estimate when we used adjusted relative risks). Among women (nine studies),^17^ ^32^ ^36^ ^39^ ^47^ ^55^ ^58^ ^67^ ^70^ the pooled adjusted relative risks were 2.19 and 3.95 for one and 20 cigarettes per day; and one cigarette per day represents 38% of the excess relative risk for 20 cigarettes per day. The pooled relative risks for the other nine studies that did not allow for multiple confounders were 1.26 and 2.11 for one and 20 cigarettes per day, and the excess relative risk for one cigarette per day was 25% (again, lower than the estimate when we used adjusted relative risks).

Among men, there were six studies of stroke,^28^ ^40^ ^42^ ^48^ ^51^ ^60^ and the pooled adjusted relative risks were 1.30 and 1.56 for smoking one and 20 cigarettes per day, with one cigarette per day representing 64% of the excess relative risk for 20 cigarettes per day. In the other 11 studies that did not allow for multiple confounders, the pooled relative risks were 1.20 and 1.68 for one and 20 cigarettes per day, and one cigarette per day had 38% of the excess relative risk for 20 cigarettes per day. Among women (five studies),^28^ ^38^ ^40^ ^47^ ^55^ the relative risks for stroke were 1.46 and 2.42 for one and 20 cigarettes per day, and one cigarette per day had 36% of the excess relative risk for 20 cigarettes per day. In the other five studies without multiple adjustment, the relative risks were 1.15 and 1.94 (15% of the excess relative risk).

All of the studies that reported results for men and women combined had relative risks adjusted for multiple confounders. Estimates of excess relative risk associated with one cigarette per day were 47% (coronary heart disease), 54% (stroke), and 51% (cardiovascular disease), from the meta-regressions in table 1. As with previous analyses, the adjusted relative risks among women for smoking one cigarette per day were higher than for men (2.19 *v* 1.74 for coronary heart disease and 1.46 *v* 1.30 for stroke) (table 2).

### Study quality

Study quality is difficult to assess, particularly when examining old studies, because “positive” design attributes were often not reported in publications. Our aim was not to examine a new association between a risk factor and a disorder but rather to use a feature of an already established causal relation, so the question of study quality is not so relevant. However, the variability in different observational study designs is the reason why we focused only on prospective cohort studies. Nevertheless, we examined study quality with the Newcastle-Ottawa assessment scale for cohort studies,^79^ using the largest set (that is, the 26 studies of coronary heart disease in men). Of these, we considered 15 to be “good quality,” and the pooled relative risk for smoking one cigarette per day was 1.62 (1.45 to 1.82), higher than that based on all studies (relative risk 1.48); our interest was in whether it would be substantially lower.

## Discussion

We have shown that a large proportion of the risk of coronary heart disease and stroke comes from smoking only a few cigarettes. This has important consequences for smokers who believe that light smoking carries little or no harm. Our estimates for people who smoke one or five cigarettes per day represent light smoking, given that the daily habits of such smokers typically vary between one and five cigarettes per day. We have also indicated that the relative risk for smoking either one or five cigarettes per day seemed to be higher among women than men. Smoking one cigarette per day carries around 40-50% of the excess risk for developing coronary heart disease and stroke of smoking 20 cigarettes per day, and smoking five cigarettes per day has around 55-65% of the excess risk (particularly when we focused on studies that reported relative risks adjusted for multiple confounders).

The high relative risk associated with low smoking levels is seen clearly in individual cohort studies (supplementary table B). For example, in one study (42 722 people), the relative risk for coronary heart disease among men was 2.74 (one to four cigarettes per day), representing 63% of the excess relative risk for smoking 20-24 cigarettes per day (relative risk 3.75).^17^ This contrasts with the effects observed for lung cancer in the same study, with relative risks of 2.79 versus 31.69,^17^ representing 6% of the excess relative risk, consistent with a linear relation between cigarette consumption and risk—that is, 5% of the consumption associated with about 5% of the excess risk, which has also been shown in other large studies.^10^ ^55^ A recent study (290 215 US adults) showed that consistent light smoking throughout a lifetime also has a large excess risk for cardiovascular disease mortality: hazard ratio 2.78 for smoking less than one cigarette per day and 1.50 for one to 10 cigarettes per day, compared with 2.77 and 3.16 for smoking 21-30 and more than 30 cigarettes per day, respectively.^80^

We have also confirmed that low cigarette consumption is associated with a high risk of stroke. This evidence is further supported by studies of second-hand smoke in never smokers,^81^ ^82^ ^83^ ^84^ in the same way as for coronary heart disease.^12^ ^83^ In a meta-analysis of seven studies of never-smokers,^82^ the relative risks for developing stroke associated with second-hand smoke, compared with unexposed never smokers, were 1.35 (95% confidence interval 1.22 to 1.50) in all participants, 1.40 (1.09 to 1.81) among men, and 1.43 (1.28 to 1.61) among women, consistent with our results for actively smoking one cigarette per day.

Potential confounding is worth considering. Different studies adjusted for different factors, but always for at least age and sex (when men and women were analysed together), which are two important confounders for cardiovascular disease. However, heavy smokers tend to have more adverse cardiovascular risk factors than light smokers (such as higher body mass index and central adiposity and poorer diet).^85^ ^86^ ^87^ Therefore, light smokers should have characteristics that are more protective against cardiovascular disease, compared with heavier smokers. Adjusting for these other risk factors should attenuate differences in cardiovascular disease risk between light and heavy smokers, not dilute them, such that when these factors are allowed for the estimates of excess risk for one or five cigarettes per day, in relation to 20, should be even larger than when based on all studies together. This is what we found when focusing only on studies that had adjusted for multiple confounding factors (table 2).

The relative risks for coronary heart disease and stroke in our analyses are in line with that for all current smokers reported by Thun et al 2013 using several cohort studies,^62^ and they also suggest that the association between smoking and these disorders has got stronger over time. For coronary heart disease, an earlier estimate of relative risk was 1.78 among men compared with 2.50 in more recent cohort studies, with similar figures for women (2.0 previously and now 2.86). However, some of this effect could be due to decreasing exposure to second-hand smoke in the reference group (never smokers) after the introduction of smoke-free legislation. If the effect is becoming stronger, the relative risk for light smokers could now be even higher than we report, with a potentially greater percentage of excess risk in relation to heavier smokers. Although we had only summary data (hence limited ability to show trends reliably), we saw some suggestion of a positive trend between the size of the relative risk for smoking one cigarette per day and the last calendar year of follow-up for each study: Spearman’s correlation 0.51 (P=0.008) for men and 0.21 (P=0.42) for women when we used studies of coronary heart disease, and 0.23 (P=0.39) and 0.56 (P=0.11) among men and women for studies of stroke.

Owing to the large effect of tobacco smoke at low doses, exposure to second-hand smoke in the reference group (never smokers) might lead to underestimation of the relative risk for one and 20 cigarettes per day and consequently dilute the percentage effect of one compared with 20 cigarettes per day. The extent of this depends on the degree of contamination (particularly for women who have never smoked, who might be more likely to be exposed to second-hand smoke from their husbands in earlier studies than men who never smoked) and the reliability of measuring exposure to second-hand smoke. Many of the studies started before smoke-free laws were implemented. Only one study adjusted for second-hand smoke,^32^ and the reported relative risks for coronary heart disease associated with one versus 20 cigarettes per day were 1.45 versus 1.82 in men and 2.03 versus 2.63 in women, in line with those from the meta-analyses.

### Strengths of study

Strengths of our analyses include that we combined data from 55 cohort study reports (which together contained 141 separate cohort studies), many of which were large. For example, the studies of coronary heart disease in men were together based on approximately 3.07 million participants, including more than 75 000 cases of coronary heart disease; for stroke, the total was approximately 3.53 million men, including at least 73 000 cases. Similarly, for women, the combined studies contained around 2.56 million participants, including at least 36 000 cases of coronary heart disease, with corresponding numbers of 3.78 million and 62 000 cases in studies of stroke. The meta-analyses should therefore provide sufficiently reliable estimates of relative risks associated with low and high cigarette consumption. By using only prospective cohort studies, in which smoking consumption is recorded before development of cardiovascular disease, we avoid biases associated with retrospective designs, such as case-control studies. We report results separately for three disease groups (coronary heart disease, stroke, and cardiovascular disease), each according to sex and age. We also did three types of statistical analyses. Importantly, results showed consistency between men and women, between the disease groups, and between the different forms of analysis.

### Limitations of study

Our analyses also had some limitations. Firstly, we did not have individual level data for study participants (many studies are old). A few datasets of cardiovascular disease and smoking are publicly available, but our aim was to be comprehensive and not restrict ourselves to having only a few studies. Furthermore, cigarette consumption is often recorded in categories (such as one to five and six to 10 cigarettes per day), not a specific number, so the ability to do regression modelling using whole numbers of cigarettes (rather than categories) is limited. Also, smokers are not expected to consume the same number of cigarettes each day, so using categories probably better reflects their intake. Having raw data would allow more sophisticated models between risk and consumption to be examined (with increased power for these analyses), compared with using a log-linear regression of summary data (based on only several smoking categories). However, our aim was to get sufficiently good approximate estimates of the excess risks in relation to the primary comparison: between the lowest and about 20 cigarettes per day group, rather than describe the whole dose-response range. As such, our estimates are supported by two sources of evidence (several individual studies and a potentially more sensitive dose-response model from a large study). The first source comprises the effects reported in individual studies (supplementary table B), showing a consistently high observed relative risk of coronary heart disease/stroke at the lowest cigarette consumption, relative to the highest consumption group, without using a fitted model,^17^ ^29^ ^33^ ^39^ ^50^ ^53^ ^55^ ^57^ ^63^ ^71^ ^72^ which are in line with our modelled estimates. The second source comprises the results from one of the largest studies (Cancer Prevention Study II),^10^ in which the authors fitted a non-linear model between a measure of tobacco smoke (particular matter: PM_2.5_) and the relative risk for cardiovascular disease, using their raw data. The model was: relative risk=1+(0.2685×PM_2.5_ dose^0.2730^). An inhaled PM_2.5_ dose of about 12 mg corresponds to about one cigarette per day, which produces a relative risk of 1.53 (both sexes combined), reassuringly in between our estimate of 1.48 for men and 1.57 for women (coronary heart disease) using our simpler log-linear model (and close to 1.63 for cardiovascular disease and both sexes combined). The relative risk estimate for 20 cigarettes per day from the more sophisticated model is 2.20, so one cigarette per day represents 44% of the excess relative risk ((1.53−1)/(2.20−1)), close to our estimate of 50% (cardiovascular disease both sexes; table 1). Furthermore, the Cancer Prevention Study II showed that there was no low threshold associated with a safe level of smoking in relation to cardiovascular disease risk, for which even an inhaled PM_2.5_ dose of 1 mg (one twelfth of a cigarette per day) has an expected relative risk of 1.25.^10^

Secondly, methods are available for estimating dose-response associations for meta-analyses that take into account that relative risk estimates across smoking categories are expected to be correlated within a study because they use the same reference group (never smokers in our case). One such method requires frequency counts in each exposure group and assumes that adjusted relative risks are similar to unadjusted ones.^88^ However, frequency data were not reported for many studies, and it is essential to use age adjusted relative risks because age is an important confounder for cardiovascular disease; and ideally other known confounders should also be accounted for. One main consequence of using methods such as this is that they produce wider 95% confidence intervals, which is unlikely to change our conclusions.

Thirdly, we used number of cigarettes per day, which is the most commonly reported measure, including in high profile studies.^55^ Although duration of smoking is also important when considering risk, it is highly correlated with age, which itself is a risk factor, so separating their effects can be difficult^89^; however, large studies tend to show a relation between duration and risk.^89^ Because light smoking seems to have dramatic effects on cardiovascular disease, shorter duration might also be associated with a higher than expected risk. This was confirmed in three cohort studies that reported duration,^38^ ^50^ ^90^ and Pope et al 2011 concluded that the steep association with cigarettes per day did not materially change when duration was allowed for in the Cancer Prevention Study II study.^10^ In another study,^50^ the relative risk for less than 10 years’ smoking duration was 1.73, compared with 2.51 for 30-40 years’ duration, representing 48% of the excess relative risk (and these relative risks had been adjusted for number of cigarettes smoked per day). Similarly, the relative risk for smoking one to five cigarettes per day was 1.88, representing 40% of the excess relative risk for smoking 15-20 cigarettes per day (3.20), and these relative risks had been adjusted for duration (years) of smoking. Although long duration has persistent cumulative effects, a large proportion of the risk seems to occur in the short term.^91^

Fourthly, some heavy smokers could misreport as light smokers at baseline (or vice versa, although few like this are expected), but if this represented a substantial proportion there would probably be non-linear associations between consumption and the risk of other disorders (for example, lung cancer), which is generally not seen in large studies.^10^ ^17^ ^55^ However, self reported smoking status has been shown to be acceptable, at least in older observational studies.^92^ Even if we assumed that misclassification was so extreme that it halved the excess risk for coronary heart disease for one cigarette per day (from table 1, 24% for men where relative risk=1.48 and 29% for women where relative risk=1.57), these estimates would still be substantially higher than the 5% expected if assuming a linear relation with risk.

### Supporting biological mechanisms

Substantial biological evidence shows that components of cigarette smoke lead to endothelial injury, cell dysfunction, atherosclerosis and acute thrombosis, and decreased ability of the blood to carry oxygen.^84^ ^89^ Several such studies were summarised previously with regards to increased platelet aggregation and increased carotid arterial wall thickening at low cigarette consumption, and coronary heart disease and stroke may have common underlying pathways.^12^ ^84^ Harmful effects at low doses are further supported by studies of second-hand smoke that show adverse actions on subclinical vascular disease and thickening of carotid artery walls.^89^ Barnoya and Glantz describe a wide range of potential mechanisms by using a comprehensive literature review to purport that platelet and endothelial function, arterial stiffness, atherosclerosis, oxidative stress, inflammation, heart rate variability, energy metabolism, and increased infarct size are all sensitive to second-hand smoke.^93^ They also noted that even brief exposure to second-hand smoke has notable adverse effects on these mechanisms, compared with that in active smokers. Three recent experimental studies focused on low consumption/exposure.^94^ ^95^ ^96^ In one study, 29 smokers each consumed a single cigarette, immediately after which they had a significant decrease in blood vessel output power and significant increase in blood vessel ageing level and remaining blood volume 25 minutes later, as markers of atherosclerosis.^94^ In another study, human coronary artery endothelial cells were exposed to the smoke equivalent to one cigarette, which led to activation of oxidant stress sensing transcription factor NFR2 and up-regulation of cytochrome p450, considered to have a role in the development of heart disease.^95^ These effects were not seen when heart cells were exposed to the vapour from one e-cigarette.^95^ A study exposed adult mice to low intensity tobacco smoke (two cigarettes) for one to two months and found adverse histopathological effects on brain cells.^96^

Indirect evidence for large harmful effects seen at low consumption also comes from studies reporting significantly reduced hospital admissions for cardiovascular disease shortly after the introduction of smoke-free legislation in various countries,^97^ ^98^ ^99^ ^100^ ^101^ including systematic reviews.^83^ ^102^ ^103^ One such review, based on 45 studies, showed that the risk of hospital admission was reduced by 15% for all coronary events and 16% for cerebrovascular events.^104^ The authors reported that the benefit remained with longer follow-up after the legislation was implemented, and greater risk reductions were seen with more comprehensive laws.

### Occasional smokers and reduced smoking

Limited data exist on the increase in risk among occasional or non-daily smokers. A previous study found a 50% increased risk of cardiovascular disease mortality among men in Finland who smoked occasionally.^105^ Of those who reported smoking daily or occasionally in the Smoking Toolkit Study in England, only 2% smoked less than one cigarette per day (“very light”),^106^ but just over 10% smoked on a non-daily basis.^107^ The non-daily smokers in the Smoking Toolkit Study smoked on average 5.2 cigarettes a day,^107^ so their risk is probably similar to that reported in our review.

In the results section, we outlined three large studies that reported little benefit on the risk of cardiovascular disease among heavy smokers who significantly reduced their consumption (unlike the large risk reduction for lung cancer), further supportive of a substantial effect of light smoking on cardiovascular disease. More evidence exists on the beliefs about health and reduced smoking (as opposed to quitting), in addition to the large US study mentioned in the introduction.^9^ One survey among 12-15 year old students showed that almost 60% of regular smokers believed that occasional smoking carried little or no health risks,^108^ and in another study 60% of e-cigarette users said that the reason for using e-cigarettes was to reduce cigarette consumption in order to reduce health risks.^109^ Even in a recent survey of 1602 people in France in 2014-15 (51% were former or current smokers), 34% thought that smoking up to 10 cigarettes per day carried no risk of lung cancer, and only half of respondents believed that there was no safe cigarette.^110^ Other surveys indicate that smokers perceive harm reduction associated with cigarettes marketed as “light” or “low tar,”^111^ ^112^ ^113^ ^114^ even though the scientific evidence shows no benefit. Although cutting down has clear benefits, particularly for risk of cancer, the reduction in cardiovascular disease risk is not as large as smokers might expect.

### Policy implications and future research

Individual research studies on the effects of light smoking have occasionally appeared in the media. Examples include “Even a cigarette a day is bad for your health” in the *New York Times* in December 2016 and the BBC’s “Light smoking doubles sudden death risk in women” in December 2012; governmental reports have also referred to this question.^89^ However, our paper is the first to combine results across many studies covering both coronary heart disease and stroke, making it a valuable reference that can be used to strengthen public health campaigns (including those on smoking cessation services) and to provide a strong health incentive for smokers to quit (particularly women), rather than cut down. We also hope to raise more awareness of the subject among cardiovascular health professionals, primary care physicians, and smoking cessation specialists.

Heart disease and stroke are common disorders and causes of death. In the UK, about 73 000 deaths due to coronary heart disease and 41 000 due to stroke occur each year (compared with 36 000 for lung cancer),^115^ and this is after the decline in mortality over time, mainly due to prevention and better treatments. However, the number of deaths is greatly over-shadowed by the number of events: more than 493 000 inpatient hospital episodes for coronary heart disease and 236 000 for stroke each year.^115^ This means that many more people are living with cardiovascular disease, with a major effect on their social and physical functioning, as well as time off work and use of local health services. The situation is similar in the US, with 370 000 deaths from coronary heart disease and 140 000 from stroke each year (compared with 155 000 for lung cancer), but the number of first heart attacks is 525 000 and that of first strokes is 610 000.^116^ ^117^ Fifteen to 20% of all cardiovascular disease events might be attributable to smoking, representing a substantial number of people that require care and treatment, but many events are avoidable. Thun et al, using recent US cohort study data (beginning 2000-10), indicated that given the increasing relative risks for coronary heart disease over time, about two thirds of the coronary heart disease deaths that occur in smokers could be attributable to their habit.^62^

The impact of smoking in places like China is of major interest. Although smoking prevalence in China has decreased in recent years, the absolute number of smokers is high, with an estimated 1 million deaths (all causes) due to tobacco in 2010.^118^ In a nationally representative survey in 2010, only 17% of current smokers said that they intended to quit, indicating that if Chinese smokers follow similar patterns to those in Western countries, many active smokers could be more inclined to reduce consumption rather than quit completely.^119^ The relatively low overall smoking prevalence among all Chinese women (<2%) might mask differences between those in rural and urban areas, as well as habits in younger women. In a 2008 survey of girls and women aged 14-24 years at high school or college, 4.2% of those in urban areas were current smokers, double the 1.9% seen in rural areas; and 38% of those surveyed in the urban locations did not believe that smoking increases the risk of cardiovascular disease (compared with 6% when asked about lung cancer).^120^

Quitting smoking greatly reduces the risk of cardiovascular disease, with important benefits gained soon after stopping (quicker than for cancer).^52^ ^55^ ^84^ ^89^ ^121^ Smokers can use nicotine containing products such as gum, patches, and electronic cigarettes. Although e-cigarettes have had much attention, they are considered by several experts to be significantly safer than cigarettes,^122^ ^123^ and they are believed to be partly responsible for the decline in smoking prevalence in the UK,^124^ findings that are in contrast to the claim that e-cigarettes help to maintain smoking rates. Therefore, they are an important component of harm reduction that can help people to quit completely,^4^ ^84^ which is necessary to significantly reduce the risk of cardiovascular disease. Although specific adverse effects of e-cigarettes on the cardiovascular system could be investigated further,^125^ ^126^ such effects, if they exist, are unlikely to be as harmful as the high risk of cardiovascular disease associated with light smoking that we show here.

### Conclusions

Smokers who cut down the number of cigarettes they use can benefit from large reductions in the risk of cancer and some benefits on cardiovascular disease. However, smoking only one to five cigarettes per day is associated with a risk of coronary heart disease and stroke that is substantially higher than many health professionals or smokers recognise (as much as half the risk of smoking 20 per day). We show clearly that no safe level of smoking exists for cardiovascular disease at which light smokers can assume that continuing to smoke does not lead to harm. Smokers need to quit completely rather than cut down if they wish to avoid most of the risk associated with heart disease and stroke, two common and major disorders caused by smoking.

**What is already known on this topic**

Smoking increases the risk of developing coronary heart disease and stroke
Many smokers believe that cutting down the number of cigarettes they smoke substantially reduces their risk of developing tobacco related disorders
Occasional individual studies and a meta-analysis of five studies in 1997 reported that light cigarette smoking (less than five per day) is associated with a higher than expected risk of coronary heart disease

**What this study adds**

Men who smoke about one cigarette per day have a 48% higher risk of heart disease than never smokers and a 25% higher risk of stroke (or 74% and 30%, respectively, when allowing for confounding factors)
The estimates are higher in women: 57% for heart disease and 31% for stroke (or 119% and 46% when allowing for multiple confounders), again compared with never smokers.
People who smoke about one cigarette each day have about 40-50% of the excess risk associated with smoking 20 per day (coronary heart disease and stroke)

## Web Extra

Extra material supplied by the author

## Notes

Ethical approval: Not needed.

Data sharing: No additional data available.

Transparency declaration: AH affirms that the manuscript is an honest, accurate, and transparent account of the study being reported; that no important aspects of the study have been omitted; and that any discrepancies from the study as planned (and, if relevant, registered) have been explained.
