# Supplementary material for: Low cigarette consumption and risk of coronary heart disease and stroke: meta-analysis of 141 cohort studies in 55 study reports
Source: BMJ. 2018 Jan 24;360:j5855. doi: 10.1136/bmj.j5855 (PMC5781309; doi:10.1136/bmj.j5855)
Supplement: Supplementary file 1 — Supplementary figures and tables [file haca040906.ww1.pdf]

Keywords used to identify MEDLINE papers were: (i) 'heart disease' OR 'cardiovascular' OR 'coronary' OR 'myocardial infarction' OR 'heart attack' for coronary heart/cardiovascular disease; and (ii) 'stroke' OR 'cerebral' OR 'cerebrovascular' OR 'cerebrovascular infarct$' OR 'haemorrhage' OR 'hemorrhage' OR 'aneurism' OR 'aneurysm' OR 'cerebral infarct$' for stroke. These were combined with smok$ AND (cohort OR prospective OR longitudinal).

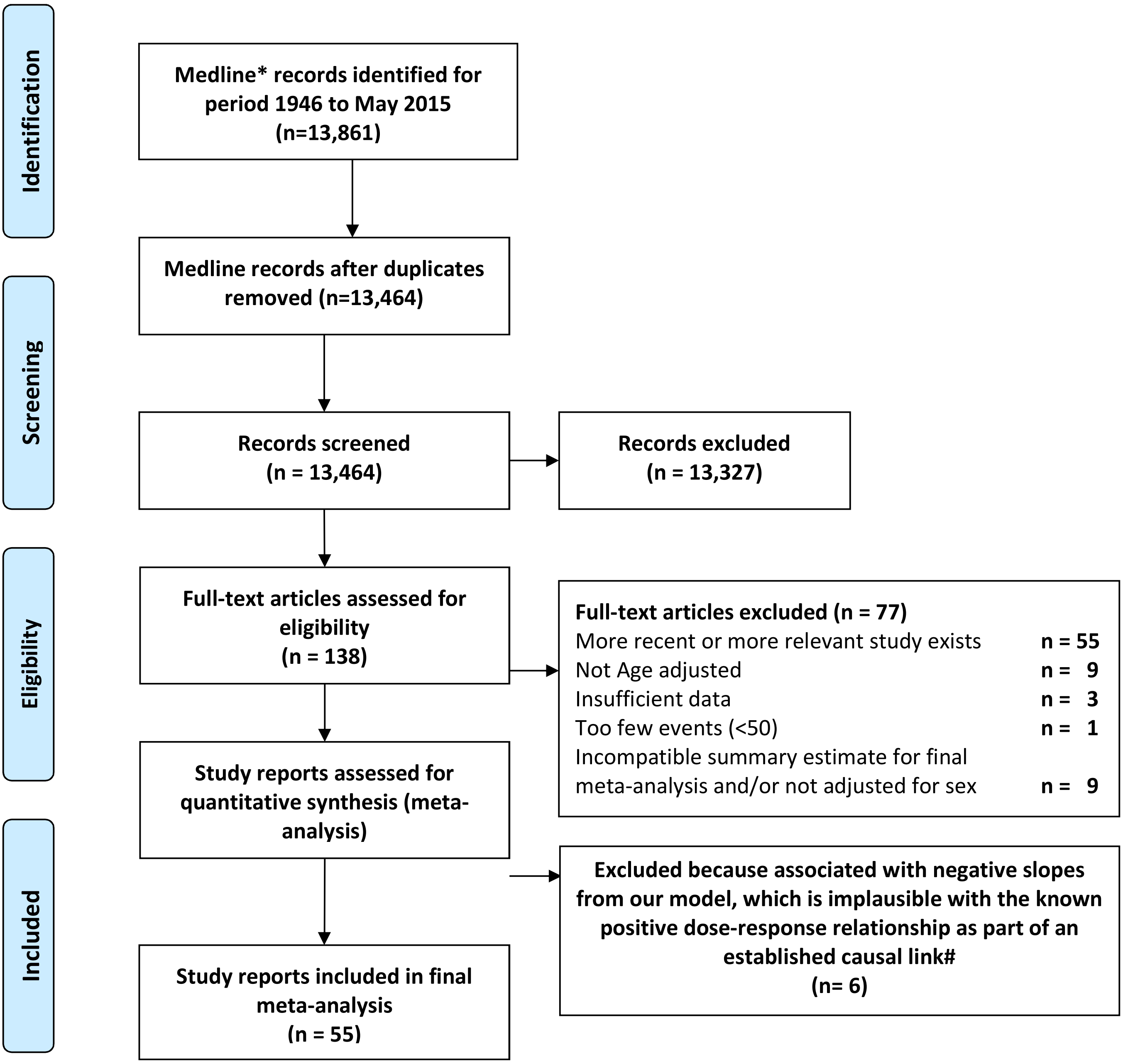

Supplementary Figure A. Selection of study reports for inclusion in the meta-analyses

- *The Health Consequences of Smoking: A Report of the Surgeon General.* 2004, contained 251 references of which 3 were included in our meta-analysis
- For multiple publications of the same study, the most recent one was used (especially if it had a larger number of CVD events) unless the older study had more details on the dose-response relationship.
- A few studies reported a regression coefficient between cigarette consumption and risk; but these were not used because consumption would not have been adjusted for extent of inhalation (using carboxyhaemoglobin and cotinine), i.e. lower inhalation with increasing cigarette consumption.[14]
- Some studies might have been missed at random if details of dose-response were only in an online appendix and not obvious from the main text.
- Within some of the 55 study reports, occasionally a specific analysis of males or females for either CHD, stroke or CVD produced a negative regression slope, and so was excluded.#

Justification for not including these:

Studies have negative slopes when the reported hazard ratios (relative risks) show a *decreasing* trend as cigarette consumption increases. Studies with negative slopes will always have a RR for 1 cigarette per day (CPD) exceeding that for 20 CPD. Therefore, including these studies would bias the results in favour of the conclusions we reach, i.e. a higher excess relative risk (RR) for smoking 1 (or 5) CPD, when expressed as a percentage of smoking 20 CPD. For example, in Rosengren et al 1992,[71] the observed relative risks are 2.8, 2.8, 3.1 and 2.1 for smoking 1-4, 5-14, 15-24 and >24 CPD. The modelled relative risks for smoking 1 or 20 CPD are 2.89 and 2.79, so the percentage of excess RR for 1 CPD is high, 106% ([2.89-1]/[2.79-1]) – compared to the average estimate for CHD of 46% among men (Table 1 of the main paper).
Observed decreasing trends could be due to chance, having a relatively small number of people or events in the lowest or highest consumption group, or fluctuating hazard ratios/relative risks; and are biologically implausible given the dose-response relationship as part of the established causal association.

5 cigarettes per day 20 cigarettes per day

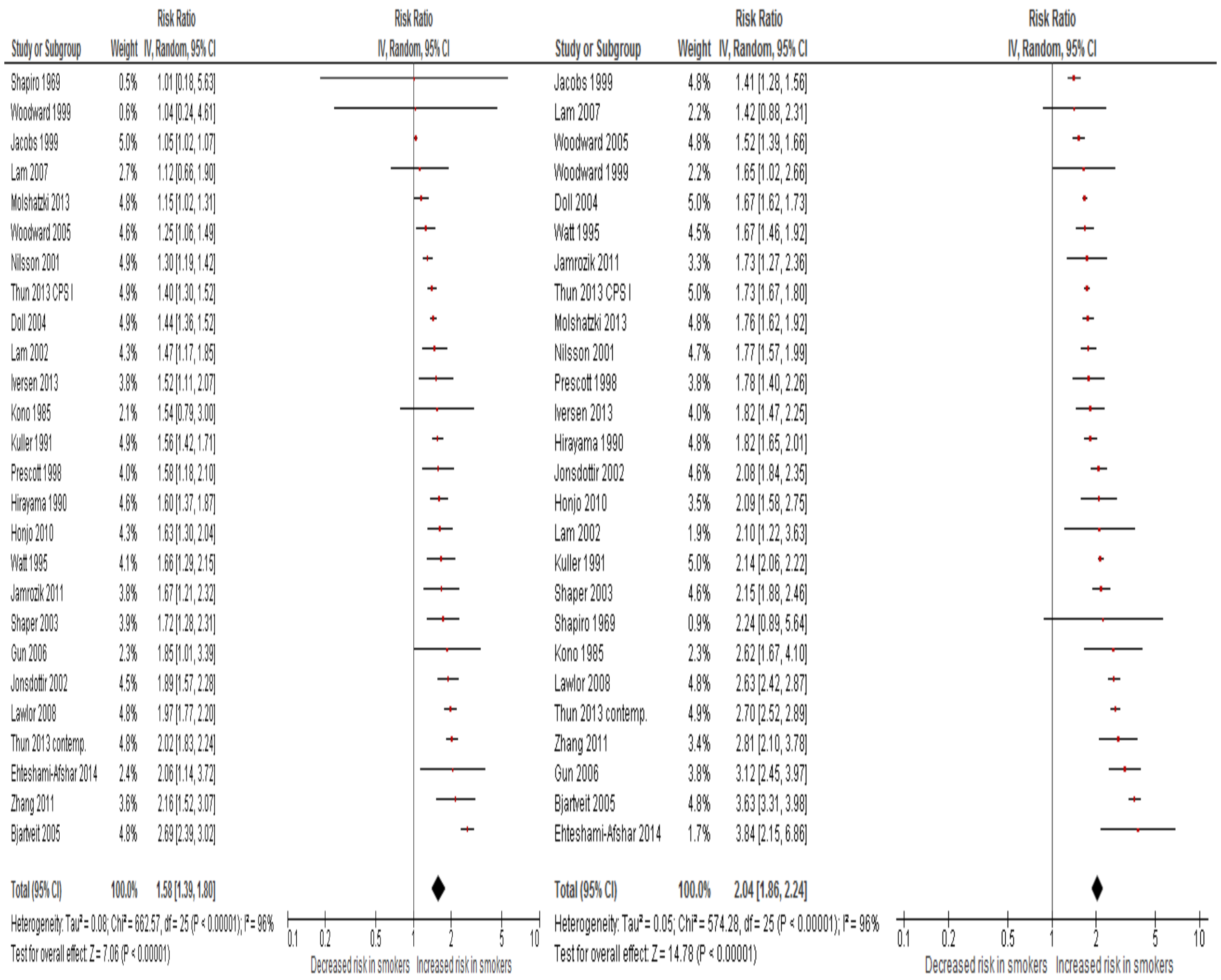

Supplementary Figure B. Forest plots for coronary heart disease, and the age-adjusted relative risks associated with smoking 5 or 20 cigarettes per day, among males. They illustrate the RRs across studies <u>in each smoking category</u>. Although these do not reflect within-study analyses, they are close to those obtained from a meta-regression (which are based on within-study analyses). Studies are in reference numbers 16 to 70.

5 cigarettes per day

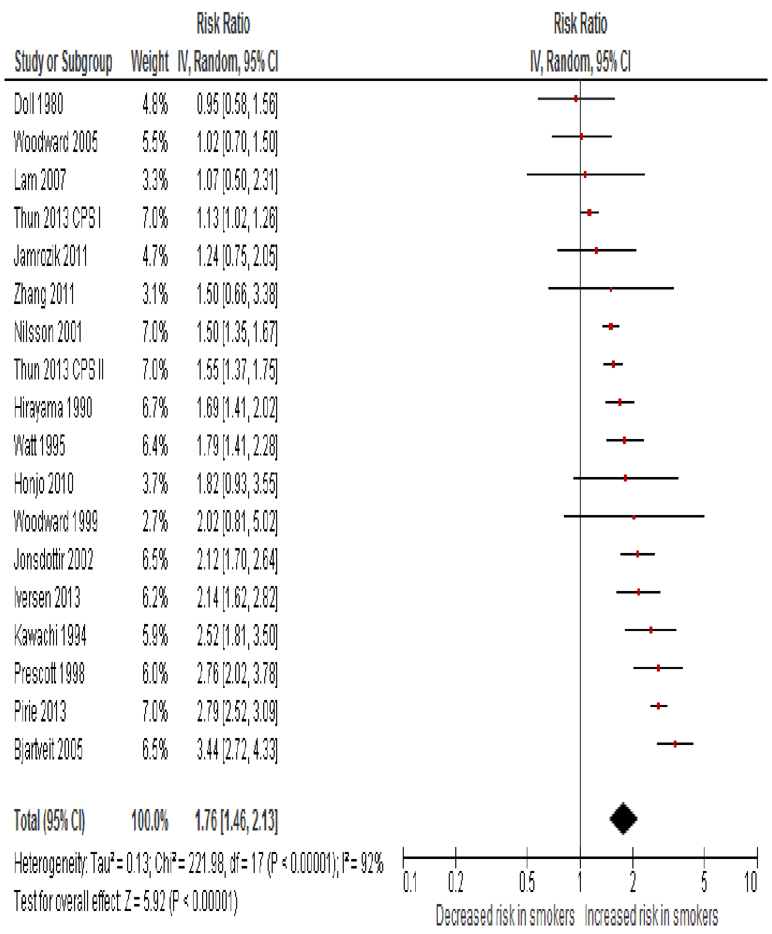

20 cigarettes per day

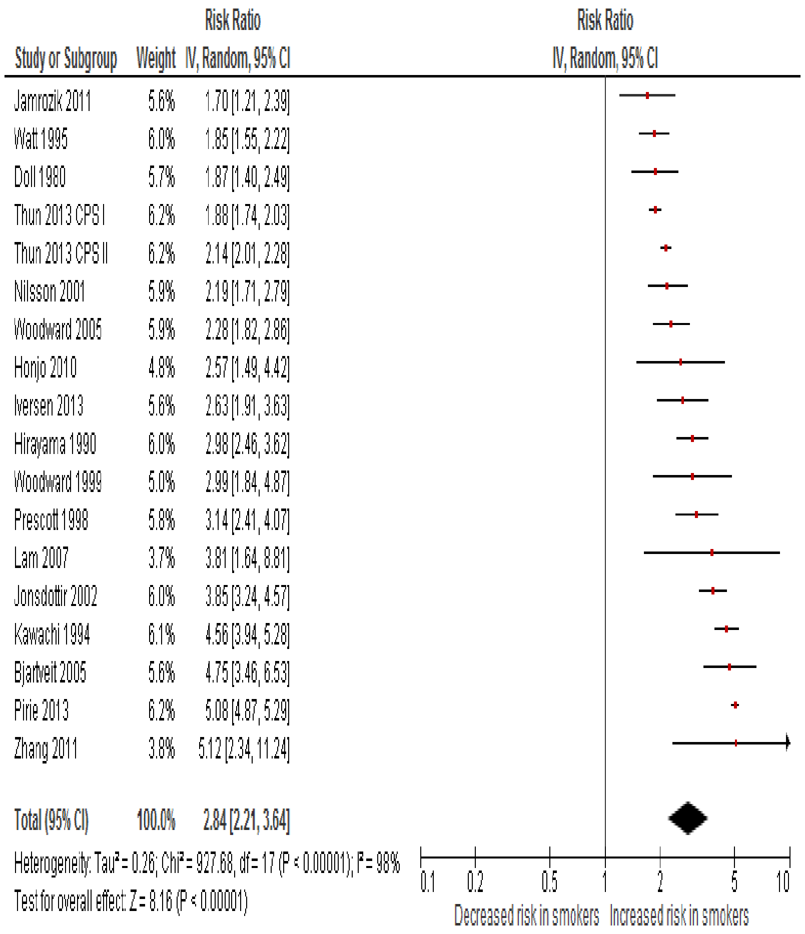

Supplementary Figure C. Forest plots for coronary heart disease, and the age-adjusted relative risks associated with smoking 5 or 20 cigarettes per day, among females. They illustrate the RRs across studies <u>in each smoking category</u>. Although these do not reflect within-study analyses, they are close to those obtained from a meta-regression (which are based on within-study analyses). Studies are in reference numbers 16 to 70.

1 cigarette per day

Study or Subgroup	Weight	Risk Ratio IV, Random, 95% CI
Gellert 2013	1.6%	1.43 [0.79, 2.57]
Mons 2015	20.9%	1.61 [1.36, 1.89]
Pope 2009	73.8%	1.66 [1.52, 1.81]
Liaw 1998	2.1%	1.72 [1.02, 2.90]
Merry 2011	1.6%	1.94 [1.08, 3.48]
**Total (95% CI)**	**100.0%**	**1.65 [1.53, 1.78]**

Heterogeneity: $Tau^2 = 0.00$; $Chi^2 = 0.68$, df = 4 (P = 0.95); $I^2 = 0\%$
Test for overall effect: Z = 13.13 (P < 0.00001)

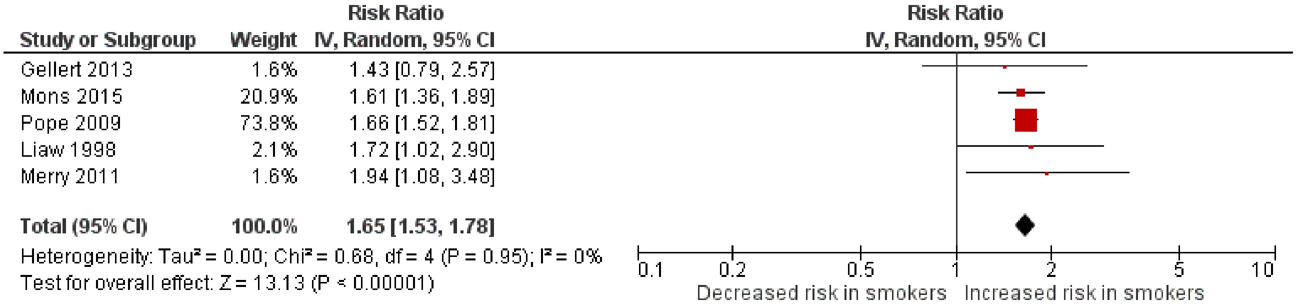

5 cigarettes per day

Study or Subgroup	Weight	Risk Ratio IV, Random, 95% CI
Gellert 2013	1.7%	1.68 [1.06, 2.66]
Mons 2015	21.8%	1.71 [1.51, 1.95]
Pope 2009	72.7%	1.72 [1.60, 1.84]
Liaw 1998	2.1%	1.84 [1.22, 2.76]
Merry 2011	1.7%	2.19 [1.39, 3.44]
**Total (95% CI)**	**100.0%**	**1.72 [1.62, 1.83]**

Heterogeneity: $Tau^2 = 0.00$; $Chi^2 = 1.19$, df = 4 (P = 0.88); $I^2 = 0\%$
Test for overall effect: Z = 17.87 (P < 0.00001)

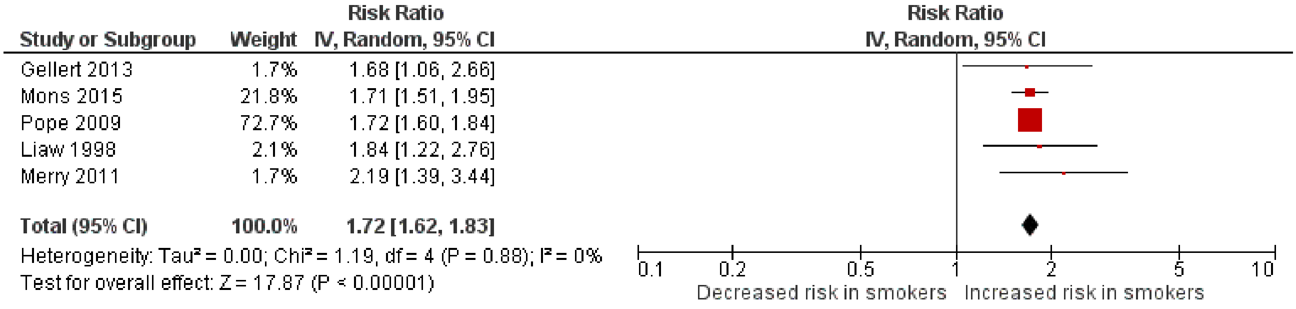

20 cigarettes per day

Study or Subgroup	Weight	Risk Ratio IV, Random, 95% CI
Pope 2009	34.1%	1.93 [1.86, 2.00]
Mons 2015	29.2%	2.16 [1.90, 2.46]
Liaw 1998	9.5%	2.33 [1.43, 3.80]
Gellert 2013	15.1%	3.07 [2.18, 4.32]
Merry 2011	12.1%	3.42 [2.27, 5.16]
**Total (95% CI)**	**100.0%**	**2.34 [1.96, 2.79]**

Heterogeneity: $Tau^2 = 0.02$; $Chi^2 = 17.10$, df = 4 (P = 0.002); $I^2 = 77\%$
Test for overall effect: Z = 9.41 (P < 0.00001)

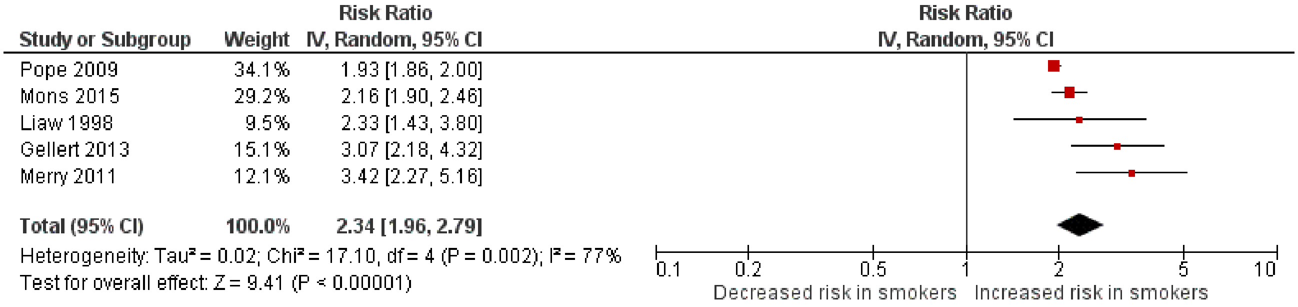

Supplementary Figure D. Forest plots for coronary heart disease and the age- and sex-adjusted relative risks associated with smoking 1, 5 or 20 cigarettes/day (for studies that did not separate males and females)

1 cigarette per day

Study or Subgroup	Weight	Risk Ratio IV, Random, 95% CI
Kahn 1966	7.7%	0.69 [0.31, 1.55]
Ragland 1988	5.6%	0.75 [0.28, 2.02]
Freund 1993	11.0%	1.13 [0.62, 2.05]
Khang 2008	18.6%	1.65 [1.21, 2.23]
Wen 2004	10.7%	1.75 [0.94, 3.25]
Burns 1997	22.2%	1.91 [1.60, 2.27]
Pooling Project Research Group 1978	5.8%	2.20 [0.83, 5.84]
Weir 1970	18.3%	2.84 [2.07, 3.89]
**Total (95% CI)**	**100.0%**	**1.65 [1.26, 2.16]**

Heterogeneity: Tau² = 0.08; Chi² = 19.49, df = 7 (P = 0.007); I² = 64%
Test for overall effect: Z = 3.64 (P = 0.0003)

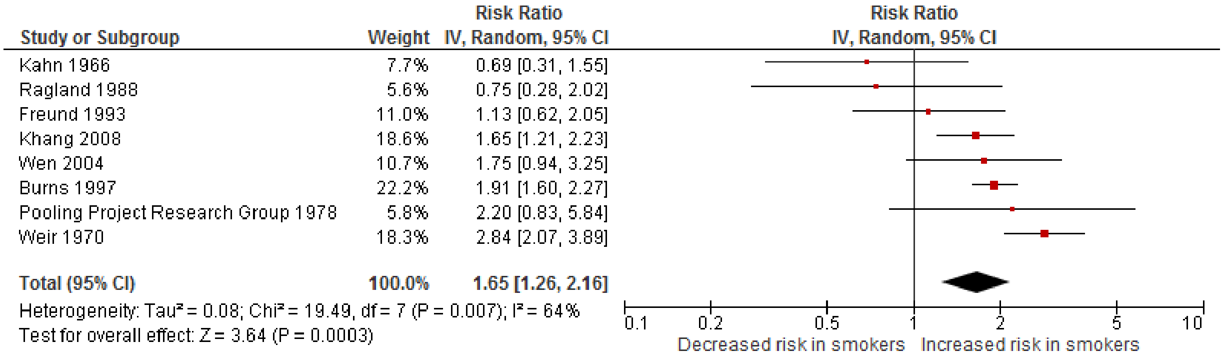

5 cigarettes per day

Study or Subgroup	Weight	Risk Ratio IV, Random, 95% CI
Kahn 1966	8.7%	0.81 [0.42, 1.56]
Ragland 1988	6.7%	0.92 [0.41, 2.07]
Freund 1993	11.8%	1.29 [0.80, 2.09]
Khang 2008	17.4%	1.74 [1.37, 2.22]
Wen 2004	12.0%	1.96 [1.22, 3.14]
Burns 1997	19.3%	2.15 [1.86, 2.47]
Pooling Project Research Group 1978	7.1%	2.59 [1.19, 5.65]
Weir 1970	16.9%	3.11 [2.40, 4.04]
**Total (95% CI)**	**100.0%**	**1.81 [1.40, 2.33]**

Heterogeneity: Tau² = 0.08; Chi² = 27.44, df = 7 (P = 0.0003); I² = 74%
Test for overall effect: Z = 4.54 (P < 0.00001)

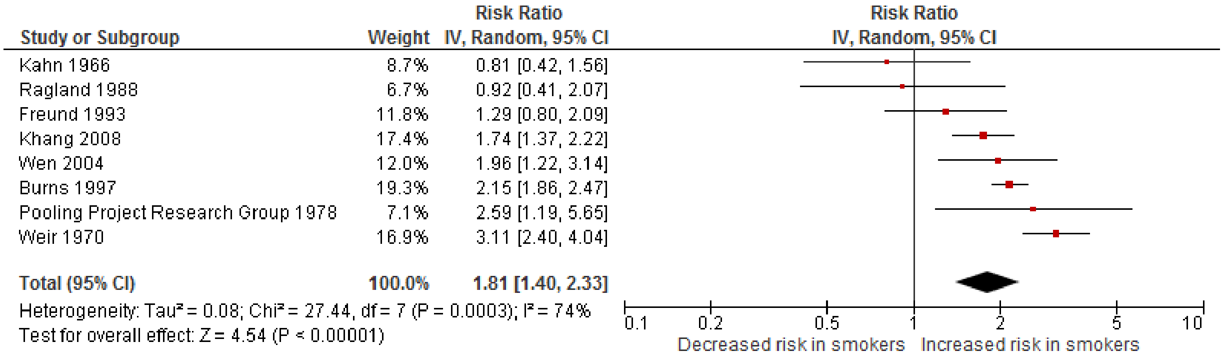

20 cigarettes per day

Study or Subgroup	Weight	Risk Ratio IV, Random, 95% CI
Kahn 1966	10.5%	1.45 [0.97, 2.18]
Ragland 1988	12.2%	2.02 [1.49, 2.74]
Khang 2008	14.3%	2.16 [1.83, 2.56]
Freund 1993	13.2%	2.16 [1.70, 2.76]
Wen 2004	10.2%	2.97 [1.95, 4.53]
Burns 1997	15.3%	3.35 [3.16, 3.56]
Weir 1970	14.6%	4.39 [3.80, 5.07]
Pooling Project Research Group 1978	9.6%	4.82 [3.03, 7.65]
**Total (95% CI)**	**100.0%**	**2.72 [2.16, 3.43]**

Heterogeneity: Tau² = 0.09; Chi² = 77.74, df = 7 (P < 0.00001); I² = 91%
Test for overall effect: Z = 8.49 (P < 0.00001)

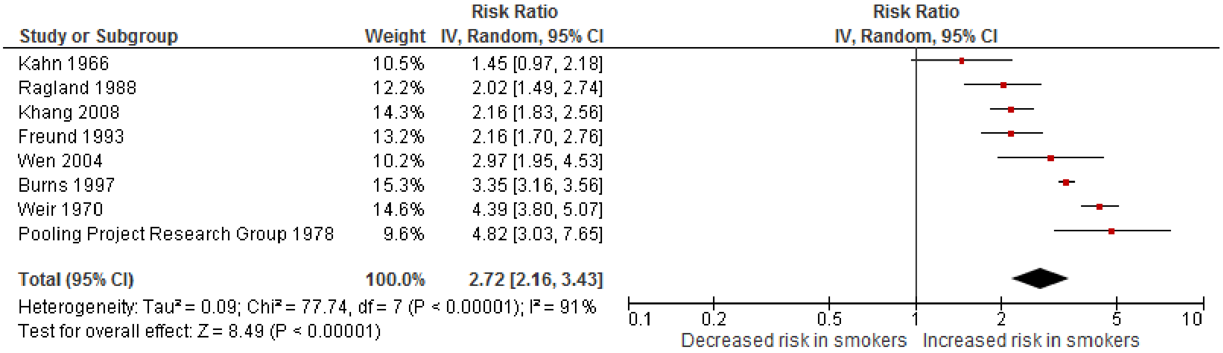

Supplementary Figure E1. Forest plots for coronary heart disease, and the relative risks associated with smoking 1, 5, 20 cigarette per day, among males aged 45 years

1 cigarette per day

Study or Subgroup	Weight	Risk Ratio IV, Random, 95% CI
Kahn 1966	9.7%	0.82 [0.52, 1.30]
Ragland 1988	3.6%	0.83 [0.34, 2.05]
Freund 1993	12.1%	0.96 [0.66, 1.39]
Pooling Project Research Group 1978	10.0%	1.42 [0.91, 2.22]
Khang 2008	14.5%	1.54 [1.14, 2.09]
Burns 1997	21.6%	1.63 [1.46, 1.81]
Wen 2004	11.4%	1.69 [1.14, 2.50]
Weir 1970	17.0%	1.90 [1.50, 2.40]
**Total (95% CI)**	**100.0%**	**1.41 [1.17, 1.70]**

Heterogeneity: Tau² = 0.04; Chi² = 19.56, df = 7 (P = 0.007); I² = 64%
Test for overall effect: Z = 3.58 (P = 0.0003)

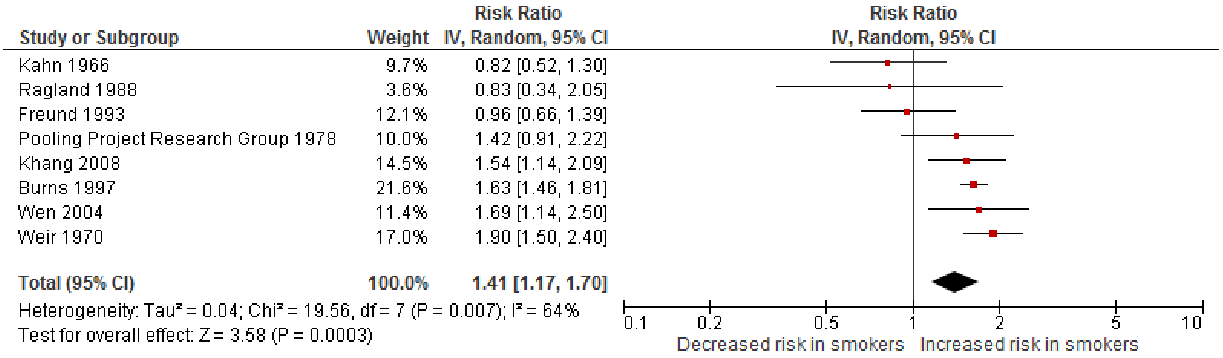

5 cigarettes per day

Study or Subgroup	Weight	Risk Ratio IV, Random, 95% CI
Kahn 1966	10.3%	0.92 [0.63, 1.34]
Ragland 1988	4.3%	0.95 [0.46, 1.98]
Freund 1993	12.5%	1.07 [0.80, 1.45]
Khang 2008	14.5%	1.63 [1.28, 2.06]
Pooling Project Research Group 1978	10.8%	1.65 [1.15, 2.35]
Wen 2004	12.4%	1.75 [1.29, 2.36]
Burns 1997	19.1%	1.77 [1.63, 1.93]
Weir 1970	16.0%	2.01 [1.66, 2.44]
**Total (95% CI)**	**100.0%**	**1.51 [1.27, 1.80]**

Heterogeneity: Tau² = 0.04; Chi² = 25.88, df = 7 (P = 0.0005); I² = 73%
Test for overall effect: Z = 4.73 (P < 0.00001)

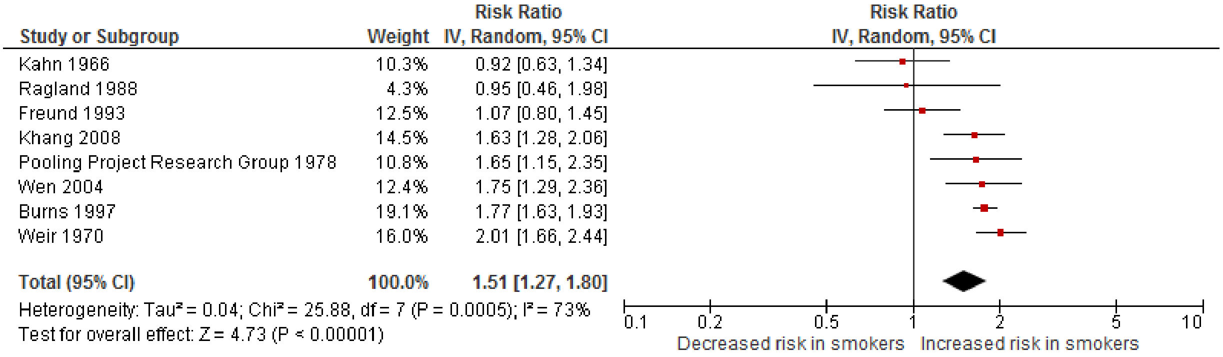

20 cigarettes per day

Study or Subgroup	Weight	Risk Ratio IV, Random, 95% CI
Kahn 1966	11.7%	1.41 [1.13, 1.76]
Ragland 1988	9.7%	1.57 [1.16, 2.12]
Freund 1993	13.7%	1.63 [1.42, 1.88]
Khang 2008	12.8%	1.98 [1.66, 2.37]
Wen 2004	10.3%	1.99 [1.51, 2.62]
Burns 1997	15.4%	2.45 [2.37, 2.54]
Weir 1970	14.4%	2.52 [2.26, 2.80]
Pooling Project Research Group 1978	12.1%	2.87 [2.33, 3.53]
**Total (95% CI)**	**100.0%**	**2.03 [1.74, 2.36]**

Heterogeneity: Tau² = 0.04; Chi² = 68.38, df = 7 (P < 0.00001); I² = 90%
Test for overall effect: Z = 9.08 (P < 0.00001)

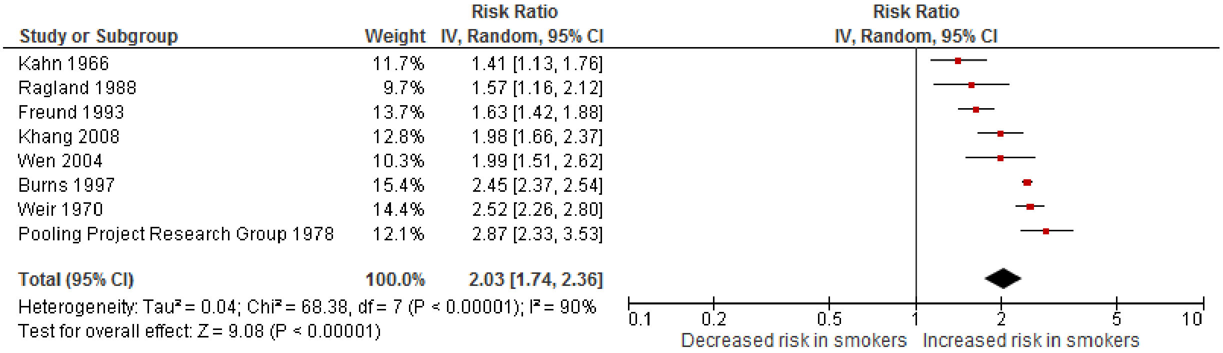

Supplementary Figure E2. Forest plots for coronary heart disease, and the relative risks associated with smoking 1, 5, 20 cigarette per day, among males aged 55 years

1 cigarette per day

Study or Subgroup	Weight	Risk Ratio IV, Random, 95% CI
Freund 1993	15.8%	0.82 [0.61, 1.10]
Pooling Project Research Group 1978	4.8%	0.92 [0.41, 2.05]
Ragland 1988	0.9%	0.93 [0.12, 7.02]
Kahn 1966	19.1%	0.97 [0.79, 1.21]
Weir 1970	15.1%	1.27 [0.92, 1.74]
Burns 1997	24.1%	1.39 [1.30, 1.47]
Khang 2008	9.5%	1.45 [0.88, 2.38]
Wen 2004	10.6%	1.63 [1.03, 2.56]
**Total (95% CI)**	**100.0%**	**1.17 [0.96, 1.43]**

Heterogeneity: $Tau^2 = 0.04$; $Chi^2 = 21.99$, df = 7 (P = 0.003); $I^2 = 68\%$
Test for overall effect: Z = 1.60 (P = 0.11)

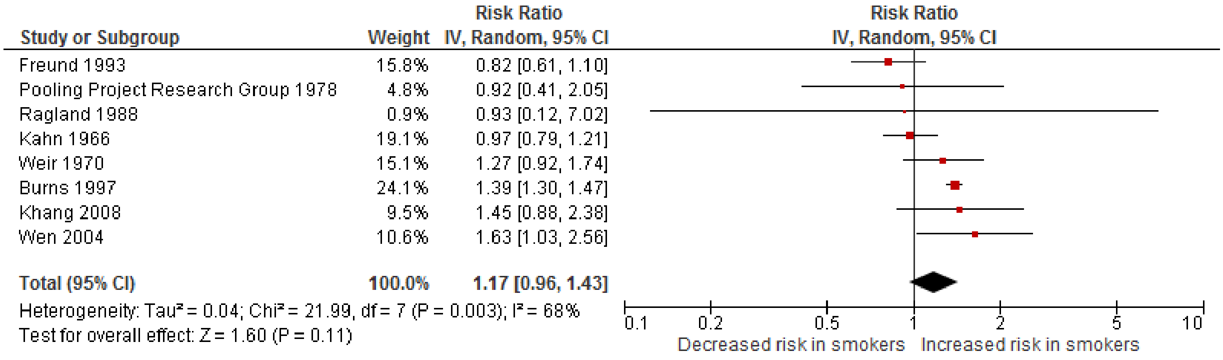

5 cigarettes per day

Study or Subgroup	Weight	Risk Ratio IV, Random, 95% CI
Freund 1993	15.8%	0.89 [0.71, 1.13]
Ragland 1988	1.1%	0.99 [0.19, 5.10]
Kahn 1966	18.0%	1.05 [0.88, 1.25]
Pooling Project Research Group 1978	5.8%	1.05 [0.55, 1.98]
Weir 1970	14.9%	1.30 [1.00, 1.69]
Burns 1997	21.3%	1.46 [1.39, 1.54]
Khang 2008	10.7%	1.52 [1.03, 2.24]
Wen 2004	12.3%	1.56 [1.11, 2.19]
**Total (95% CI)**	**100.0%**	**1.24 [1.03, 1.48]**

Heterogeneity: $Tau^2 = 0.04$; $Chi^2 = 29.56$, df = 7 (P = 0.0001); $I^2 = 76\%$
Test for overall effect: Z = 2.31 (P = 0.02)

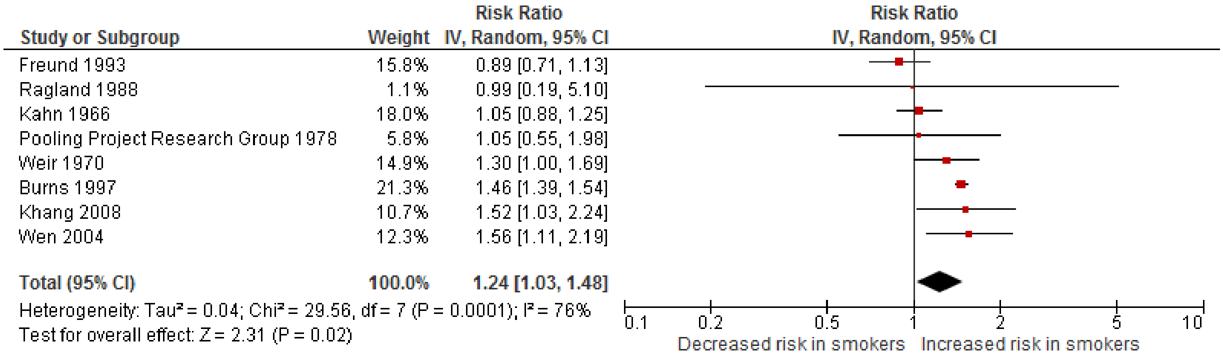

20 cigarettes per day

Study or Subgroup	Weight	Risk Ratio IV, Random, 95% CI
Ragland 1988	4.2%	1.22 [0.63, 2.36]
Freund 1993	16.0%	1.23 [1.07, 1.42]
Wen 2004	8.4%	1.33 [0.89, 1.97]
Kahn 1966	16.8%	1.37 [1.23, 1.53]
Weir 1970	15.8%	1.44 [1.25, 1.67]
Pooling Project Research Group 1978	9.3%	1.71 [1.20, 2.44]
Burns 1997	18.3%	1.80 [1.76, 1.84]
Khang 2008	11.1%	1.82 [1.35, 2.43]
**Total (95% CI)**	**100.0%**	**1.49 [1.28, 1.74]**

Heterogeneity: $Tau^2 = 0.03$; $Chi^2 = 59.01$, df = 7 (P < 0.00001); $I^2 = 88\%$
Test for overall effect: Z = 5.07 (P < 0.00001)

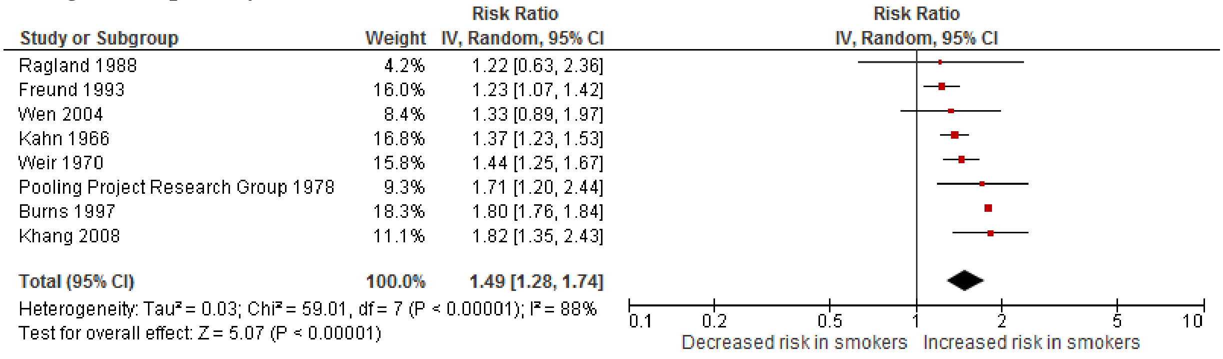

Supplementary Figure E3. Forest plots for coronary heart disease, and the relative risks associated with smoking 1, 5, 20 cigarette per day, among males aged 65 years

1 cigarette per day

Study or Subgroup	Weight	Risk Ratio IV, Random, 95% CI
Bush 1983	19.4%	0.97 [0.56, 1.65]
Freund 1993	14.5%	1.01 [0.54, 1.90]
Burns 1997	66.2%	1.43 [1.13, 1.81]
**Total (95% CI)**	**100.0%**	**1.26 [0.98, 1.62]**

Heterogeneity: $Tau^2 = 0.01$; $Chi^2 = 2.40$, $df = 2$ ($P = 0.30$); $I^2 = 17\%$
Test for overall effect: $Z = 1.78$ ($P = 0.08$)

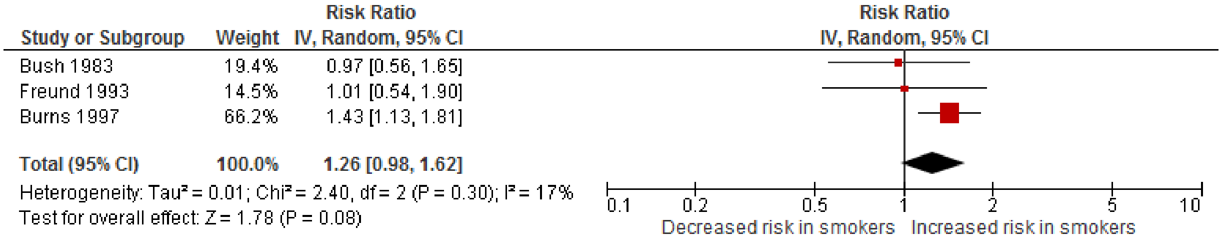

5 cigarettes per day

Study or Subgroup	Weight	Risk Ratio IV, Random, 95% CI
Freund 1993	27.0%	1.03 [0.63, 1.66]
Bush 1983	29.9%	1.15 [0.75, 1.75]
Burns 1997	43.1%	1.77 [1.47, 2.13]
**Total (95% CI)**	**100.0%**	**1.34 [0.92, 1.96]**

Heterogeneity: $Tau^2 = 0.08$; $Chi^2 = 6.69$, $df = 2$ ($P = 0.04$); $I^2 = 70\%$
Test for overall effect: $Z = 1.53$ ($P = 0.13$)

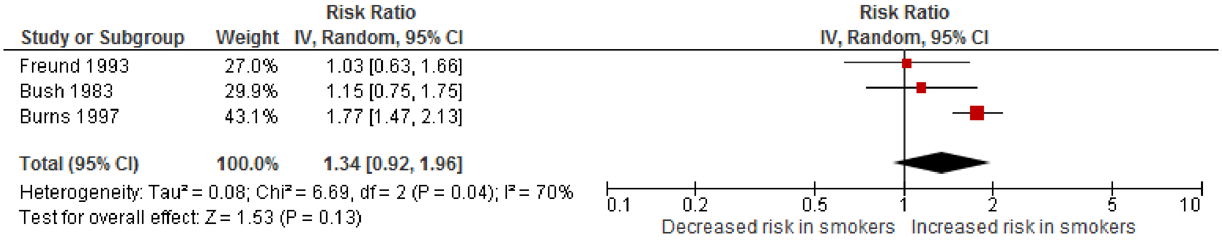

20 cigarettes per day

Study or Subgroup	Weight	Risk Ratio IV, Random, 95% CI
Freund 1993	30.8%	1.09 [0.69, 1.74]
Bush 1983	33.7%	2.19 [1.65, 2.92]
Burns 1997	35.5%	3.98 [3.56, 4.44]
**Total (95% CI)**	**100.0%**	**2.19 [1.11, 4.32]**

Heterogeneity: $Tau^2 = 0.34$; $Chi^2 = 39.71$, $df = 2$ ($P < 0.00001$); $I^2 = 95\%$
Test for overall effect: $Z = 2.25$ ($P = 0.02$)

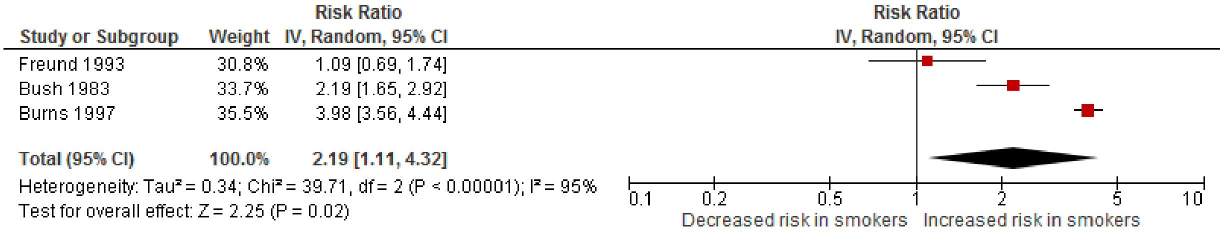

Supplementary Figure F1. Forest plots for coronary heart disease, and the relative risks associated with smoking 1, 5, 20 cigarette per day, among females aged 45 years

1 cigarette per day

Study or Subgroup	Weight	Risk Ratio IV, Random, 95% CI
Bush 1983	17.3%	1.03 [0.74, 1.43]
Freund 1993	12.5%	1.04 [0.71, 1.53]
Burns 1997	70.2%	1.29 [1.11, 1.49]
**Total (95% CI)**	**100.0%**	**1.21 [1.05, 1.39]**

Heterogeneity: $Tau^2 = 0.00$; $Chi^2 = 2.17$, $df = 2$ ($P = 0.34$); $I^2 = 8\%$
Test for overall effect: $Z = 2.62$ ($P = 0.009$)

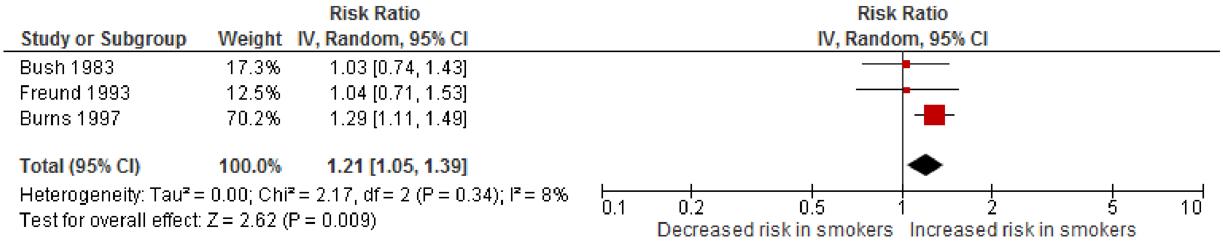

5 cigarettes per day

Study or Subgroup	Weight	Risk Ratio IV, Random, 95% CI
Freund 1993	27.8%	1.06 [0.79, 1.43]
Bush 1983	30.9%	1.13 [0.88, 1.46]
Burns 1997	41.3%	1.52 [1.36, 1.71]
**Total (95% CI)**	**100.0%**	**1.26 [0.98, 1.62]**

Heterogeneity: $Tau^2 = 0.04$; $Chi^2 = 8.01$, $df = 2$ ($P = 0.02$); $I^2 = 75\%$
Test for overall effect: $Z = 1.78$ ($P = 0.07$)

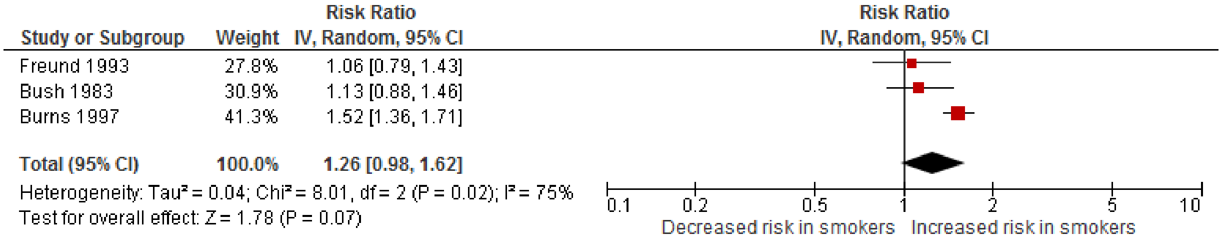

20 cigarettes per day

Study or Subgroup	Weight	Risk Ratio IV, Random, 95% CI
Freund 1993	32.1%	1.15 [0.88, 1.51]
Bush 1983	33.3%	1.61 [1.33, 1.95]
Burns 1997	34.5%	2.88 [2.70, 3.07]
**Total (95% CI)**	**100.0%**	**1.77 [1.00, 3.11]**

Heterogeneity: $Tau^2 = 0.24$; $Chi^2 = 68.55$, $df = 2$ ($P < 0.00001$); $I^2 = 97\%$
Test for overall effect: $Z = 1.98$ ($P = 0.05$)

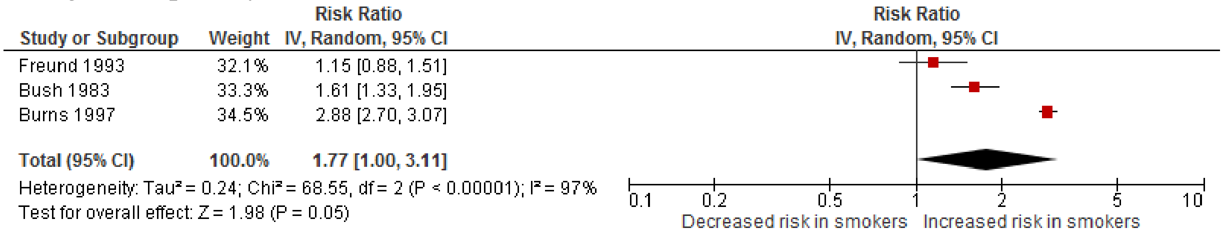

Supplementary Figure F2. Forest plots for coronary heart disease, and the relative risks associated with smoking 1, 5, 20 cigarette per day, among females aged 55 years

1 cigarette per day

Study or Subgroup	Weight	Risk Ratio IV, Random, 95% CI
Freund 1993	6.5%	1.08 [0.78, 1.48]
Bush 1983	4.4%	1.10 [0.75, 1.62]
Burns 1997	89.1%	1.16 [1.06, 1.26]
**Total (95% CI)**	**100.0%**	**1.15 [1.06, 1.25]**

Heterogeneity: Tau² = 0.00; Chi² = 0.25, df = 2 (P = 0.88); I² = 0%
Test for overall effect: Z = 3.38 (P = 0.0007)

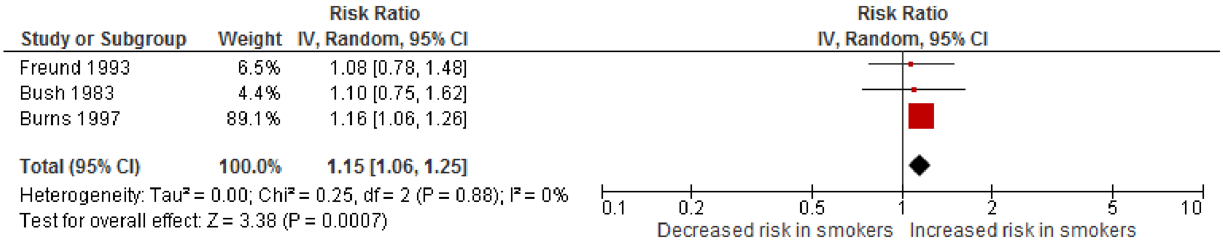

5 cigarettes per day

Study or Subgroup	Weight	Risk Ratio IV, Random, 95% CI
Freund 1993	18.8%	1.10 [0.87, 1.40]
Bush 1983	12.8%	1.12 [0.83, 1.50]
Burns 1997	68.4%	1.31 [1.23, 1.40]
**Total (95% CI)**	**100.0%**	**1.24 [1.11, 1.40]**

Heterogeneity: Tau² = 0.00; Chi² = 2.79, df = 2 (P = 0.25); I² = 28%
Test for overall effect: Z = 3.71 (P = 0.0002)

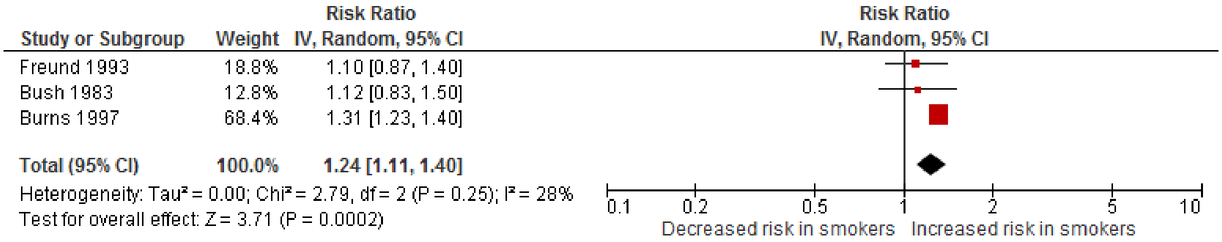

20 cigarettes per day

Study or Subgroup	Weight	Risk Ratio IV, Random, 95% CI
Bush 1983	30.9%	1.18 [0.86, 1.62]
Freund 1993	32.6%	1.22 [0.94, 1.58]
Burns 1997	36.6%	2.08 [2.00, 2.18]
**Total (95% CI)**	**100.0%**	**1.47 [0.94, 2.29]**

Heterogeneity: Tau² = 0.14; Chi² = 27.49, df = 2 (P < 0.00001); I² = 93%
Test for overall effect: Z = 1.69 (P = 0.09)

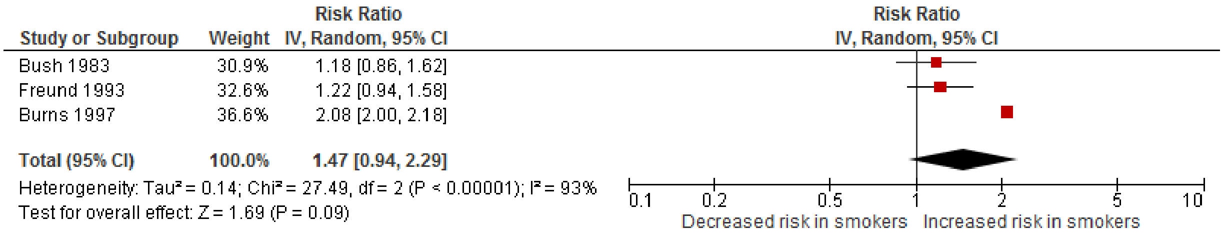

Supplementary Figure F3. Forest plots for coronary heart disease, and the relative risks associated with smoking 1, 5, 20 cigarette per day, among females aged 65 years

5 cigarettes per day

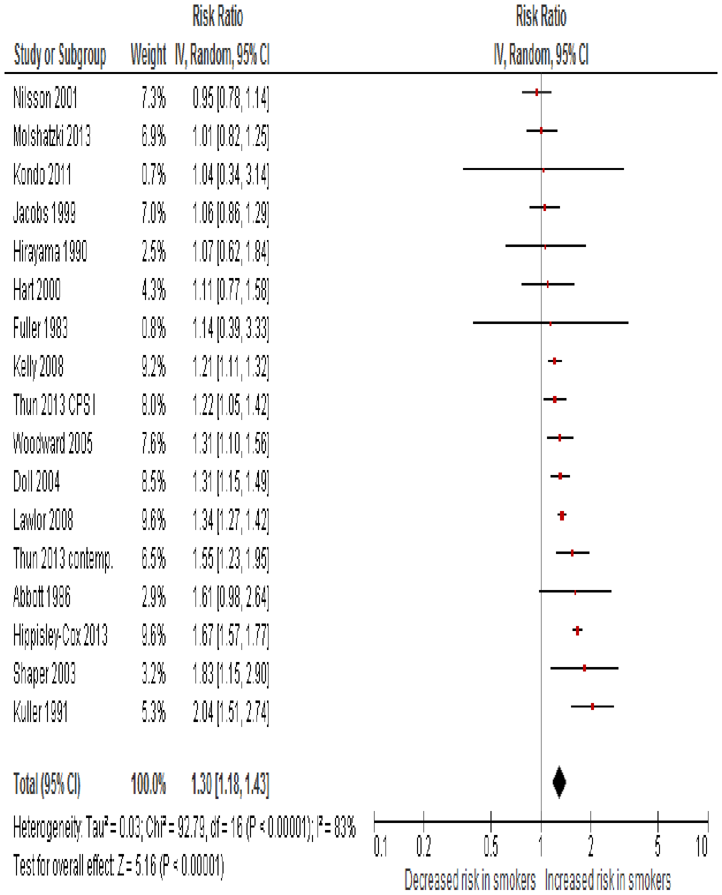

20 cigarettes per day

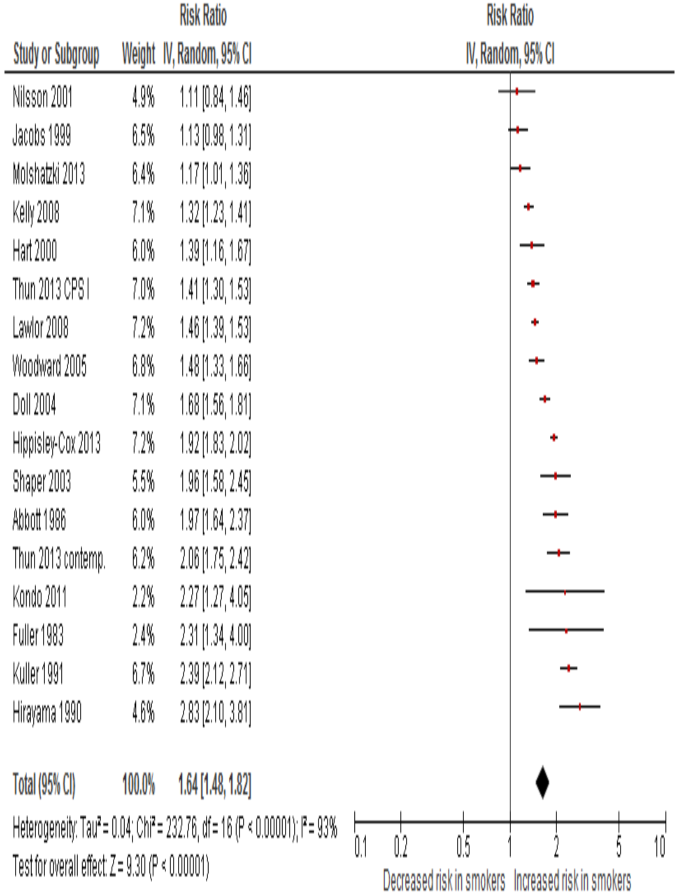

Supplementary Figure G. Forest plots for stroke, and the age-adjusted relative risks associated with smoking 5 or 20 cigarettes per day, among males.

5 cigarettes per day

Study or Subgroup	Weight	Risk Ratio IV, Random, 95% CI
Woodward 2005	7.9%	1.08 [0.74, 1.58]
Thun 2013 CPS I	11.8%	1.12 [0.95, 1.34]
Hirayama 1990	13.0%	1.15 [1.04, 1.26]
Kelly 2008	12.8%	1.19 [1.06, 1.33]
Lam 2007	4.1%	1.37 [0.69, 2.71]
Thun 2013 CPS II	11.0%	1.46 [1.17, 1.81]
Hippisley-Cox 2013	13.2%	1.58 [1.47, 1.70]
Kawachi 1993	6.7%	1.95 [1.25, 3.06]
Honjo 2010	7.1%	2.09 [1.37, 3.20]
Pirie 2013	12.5%	2.11 [1.85, 2.40]
**Total (95% CI)**	**100.0%**	**1.44 [1.22, 1.70]**

Heterogeneity: $Tau^2 = 0.05$; $Chi^2 = 87.35$, df = 9 (P < 0.00001); $I^2 = 90\%$
Test for overall effect: Z = 4.37 (P < 0.0001)

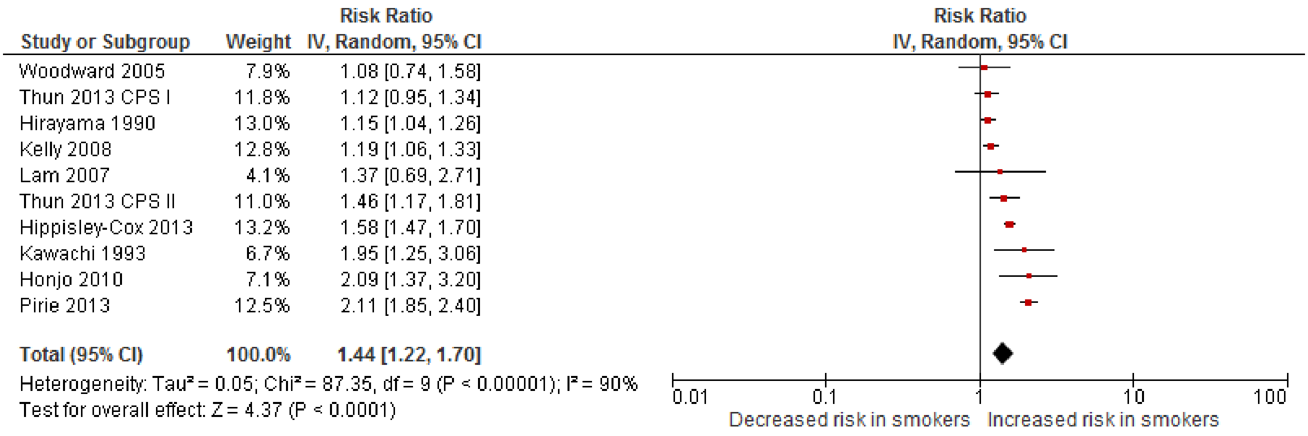

20 cigarettes per day

Study or Subgroup	Weight	Risk Ratio IV, Random, 95% CI
Hirayama 1990	10.9%	1.17 [1.02, 1.35]
Kelly 2008	11.0%	1.33 [1.16, 1.52]
Thun 2013 CPS I	11.0%	1.84 [1.63, 2.08]
Hippisley-Cox 2013	11.3%	2.08 [1.98, 2.19]
Woodward 2005	9.7%	2.17 [1.62, 2.90]
Thun 2013 CPS II	11.1%	2.39 [2.16, 2.66]
Honjo 2010	9.2%	2.61 [1.83, 3.70]
Kawachi 1993	10.5%	3.20 [2.60, 3.95]
Lam 2007	4.1%	3.21 [1.24, 8.30]
Pirie 2013	11.3%	3.51 [3.30, 3.72]
**Total (95% CI)**	**100.0%**	**2.16 [1.69, 2.75]**

Heterogeneity: $Tau^2 = 0.14$; $Chi^2 = 383.83$, df = 9 (P < 0.00001); $I^2 = 98\%$
Test for overall effect: Z = 6.21 (P < 0.00001)

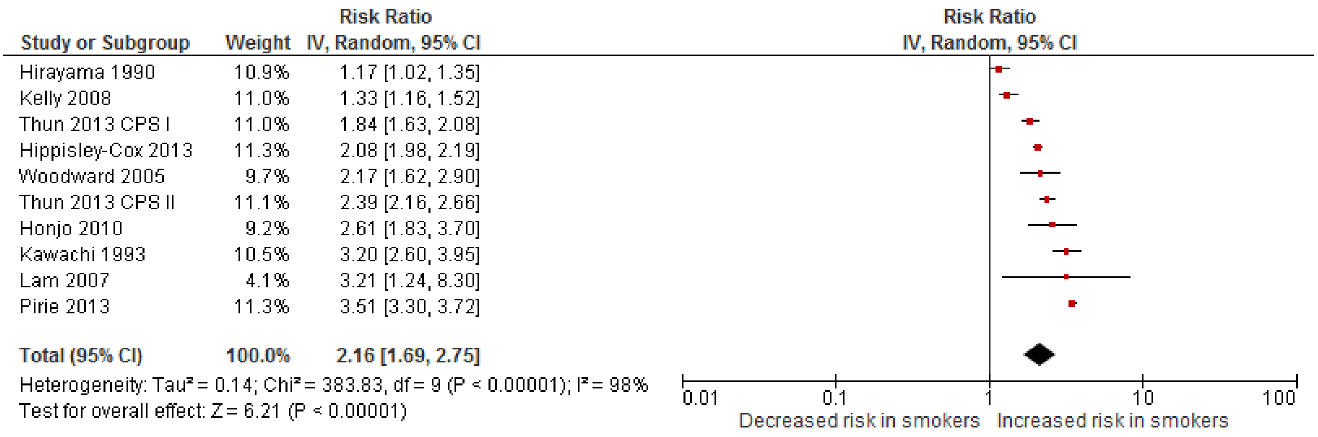

Supplementary Figure H. Forest plots for stroke, and the age-adjusted relative risks associated with smoking 5 or 20 cigarettes per day, among females

1 cigarette per day

Study or Subgroup	Weight	Risk Ratio IV, Random, 95% CI
Mons 2015	64.8%	1.35 [1.16, 1.57]
Gellert 2013	35.2%	1.90 [1.28, 2.83]
**Total (95% CI)**	**100.0%**	**1.52 [1.10, 2.10]**

Heterogeneity: Tau² = 0.04; Chi² = 2.52, df = 1 (P = 0.11); I² = 60%
Test for overall effect: Z = 2.56 (P = 0.01)

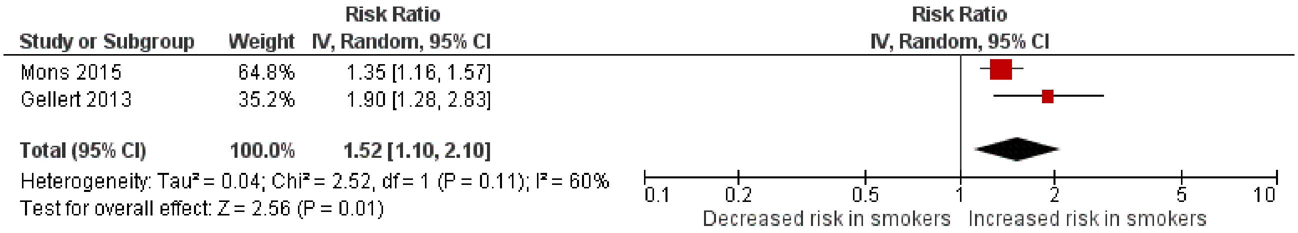

5 cigarettes per day

Study or Subgroup	Weight	Risk Ratio IV, Random, 95% CI
Mons 2015	59.9%	1.43 [1.27, 1.61]
Gellert 2013	40.1%	1.97 [1.45, 2.67]
**Total (95% CI)**	**100.0%**	**1.63 [1.19, 2.21]**

Heterogeneity: Tau² = 0.04; Chi² = 3.72, df = 1 (P = 0.05); I² = 73%
Test for overall effect: Z = 3.09 (P = 0.002)

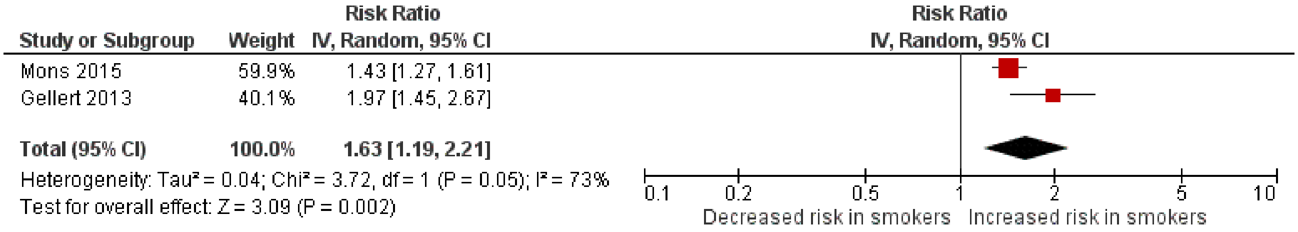

20 cigarettes per day

Study or Subgroup	Weight	Risk Ratio IV, Random, 95% CI
Mons 2015	71.2%	1.78 [1.61, 1.97]
Gellert 2013	28.8%	2.25 [1.64, 3.10]
**Total (95% CI)**	**100.0%**	**1.90 [1.54, 2.35]**

Heterogeneity: Tau² = 0.01; Chi² = 1.93, df = 1 (P = 0.17); I² = 48%
Test for overall effect: Z = 6.00 (P < 0.00001)

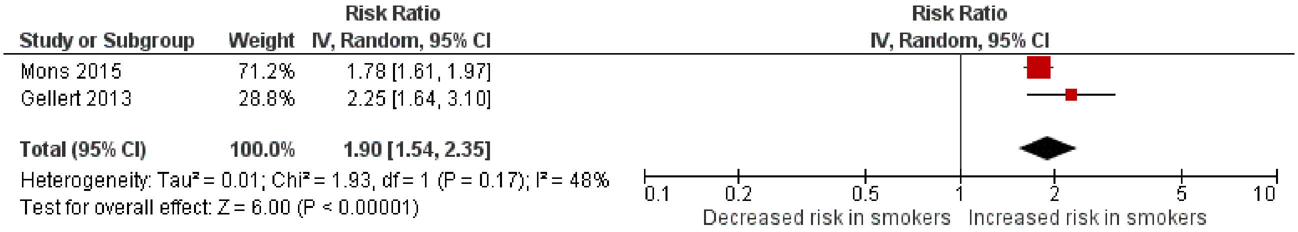

Supplementary Figure I. Forest plots for stroke and the age- and sex-adjusted relative risks associated with smoking 1, 5 or 20 cigarettes/day (for studies that did not separate males and females)

1 cigarette per day

Study or Subgroup	Weight	Risk Ratio IV, Random, 95% CI
Wen 2004	49.1%	1.31 [0.83, 2.06]
Burns 1997	50.9%	1.52 [0.97, 2.37]
**Total (95% CI)**	**100.0%**	**1.41 [1.03, 1.94]**

Heterogeneity: Tau² = 0.00; Chi² = 0.21, df = 1 (P = 0.65); I² = 0%
Test for overall effect: Z = 2.14 (P = 0.03)

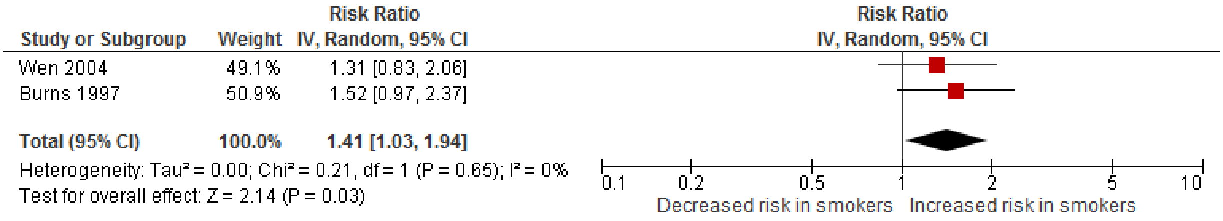

5 cigarettes per day

Study or Subgroup	Weight	Risk Ratio IV, Random, 95% CI
Wen 2004	52.2%	1.50 [1.06, 2.12]
Burns 1997	47.8%	1.77 [1.23, 2.55]
**Total (95% CI)**	**100.0%**	**1.62 [1.26, 2.09]**

Heterogeneity: Tau² = 0.00; Chi² = 0.42, df = 1 (P = 0.51); I² = 0%
Test for overall effect: Z = 3.78 (P = 0.0002)

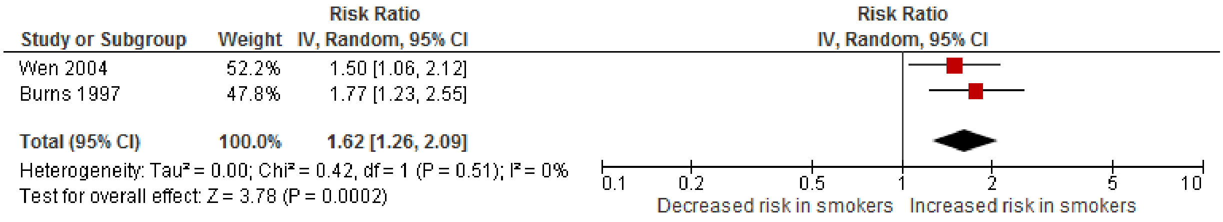

20 cigarettes per day

Study or Subgroup	Weight	Risk Ratio IV, Random, 95% CI
Wen 2004	35.6%	2.48 [1.84, 3.33]
Burns 1997	64.4%	3.15 [2.70, 3.67]
**Total (95% CI)**	**100.0%**	**2.89 [2.31, 3.62]**

Heterogeneity: Tau² = 0.01; Chi² = 1.99, df = 1 (P = 0.16); I² = 50%
Test for overall effect: Z = 9.26 (P < 0.00001)

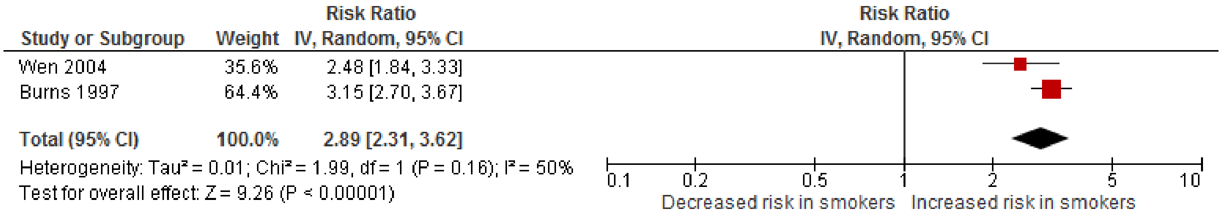

Supplementary Figure J1. Forest plots for stroke, and the relative risks associated with smoking 1, 5, 20 cigarette per day, among males aged 45 years

1 cigarette per day

Study or Subgroup	Weight	Risk Ratio IV, Random, 95% CI
Wen 2004	49.1%	1.13 [0.85, 1.51]
Burns 1997	50.9%	1.41 [1.06, 1.87]
**Total (95% CI)**	**100.0%**	**1.27 [1.02, 1.57]**

Heterogeneity: Tau² = 0.00; Chi² = 1.13, df = 1 (P = 0.29); I² = 11%
Test for overall effect: Z = 2.17 (P = 0.03)

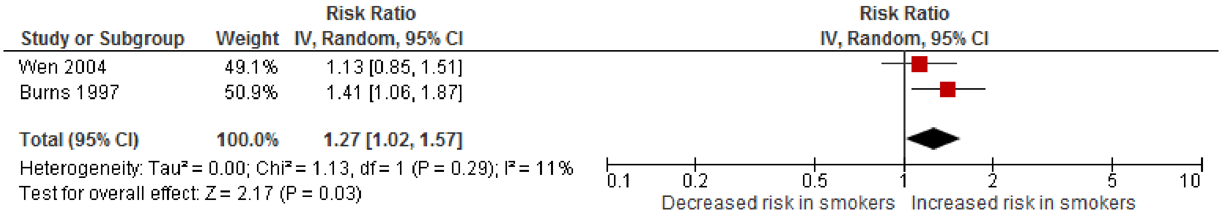

5 cigarettes per day

Study or Subgroup	Weight	Risk Ratio IV, Random, 95% CI
Wen 2004	51.0%	1.23 [0.99, 1.53]
Burns 1997	49.0%	1.57 [1.25, 1.97]
**Total (95% CI)**	**100.0%**	**1.39 [1.09, 1.75]**

Heterogeneity: Tau² = 0.02; Chi² = 2.20, df = 1 (P = 0.14); I² = 55%
Test for overall effect: Z = 2.71 (P = 0.007)

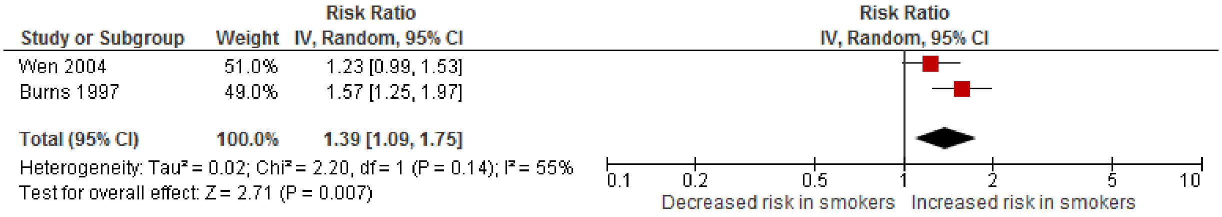

20 cigarettes per day

Study or Subgroup	Weight	Risk Ratio IV, Random, 95% CI
Wen 2004	46.6%	1.69 [1.39, 2.04]
Burns 1997	53.4%	2.34 [2.12, 2.57]
**Total (95% CI)**	**100.0%**	**2.01 [1.46, 2.76]**

Heterogeneity: Tau² = 0.05; Chi² = 8.91, df = 1 (P = 0.003); I² = 89%
Test for overall effect: Z = 4.27 (P < 0.0001)

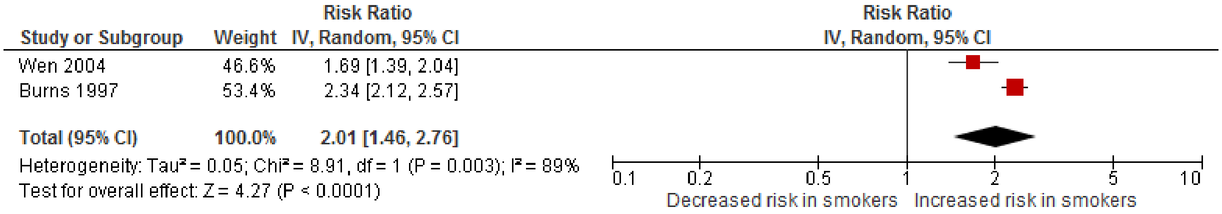

Supplementary Figure J2. Forest plots for stroke, and the relative risks associated with smoking 1, 5, 20 cigarette per day, among males aged 55 years

1 cigarette per day

Study or Subgroup	Weight	Risk Ratio IV, Random, 95% CI
Wen 2004	36.8%	0.98 [0.71, 1.36]
Burns 1997	63.2%	1.31 [1.12, 1.52]
**Total (95% CI)**	**100.0%**	**1.18 [0.90, 1.54]**

Heterogeneity: $Tau^2 = 0.02$; $Chi^2 = 2.45$, df = 1 (P = 0.12); $I^2 = 59\%$
Test for overall effect: Z = 1.17 (P = 0.24)

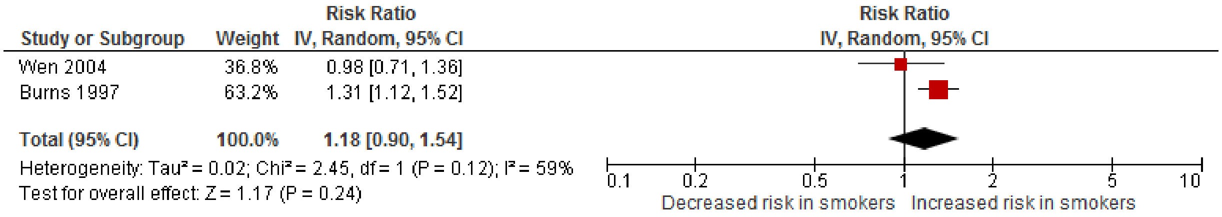

5 cigarettes per day

Study or Subgroup	Weight	Risk Ratio IV, Random, 95% CI
Wen 2004	44.2%	1.01 [0.79, 1.29]
Burns 1997	55.8%	1.39 [1.23, 1.57]
**Total (95% CI)**	**100.0%**	**1.21 [0.89, 1.64]**

Heterogeneity: $Tau^2 = 0.04$; $Chi^2 = 5.06$, df = 1 (P = 0.02); $I^2 = 80\%$
Test for overall effect: Z = 1.21 (P = 0.23)

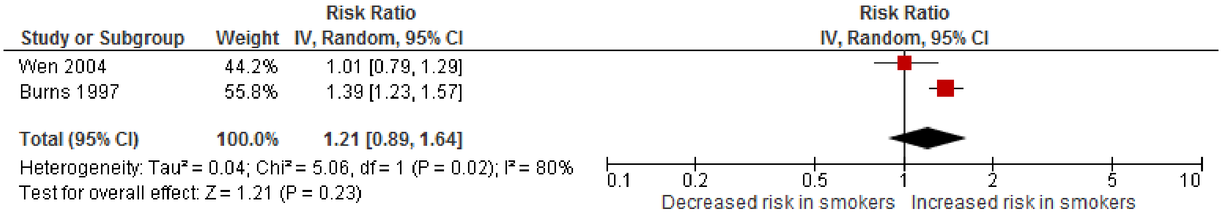

20 cigarettes per day

Study or Subgroup	Weight	Risk Ratio IV, Random, 95% CI
Wen 2004	44.8%	1.15 [0.88, 1.50]
Burns 1997	55.2%	1.73 [1.64, 1.83]
**Total (95% CI)**	**100.0%**	**1.44 [0.96, 2.15]**

Heterogeneity: $Tau^2 = 0.08$; $Chi^2 = 8.87$, df = 1 (P = 0.003); $I^2 = 89\%$
Test for overall effect: Z = 1.77 (P = 0.08)

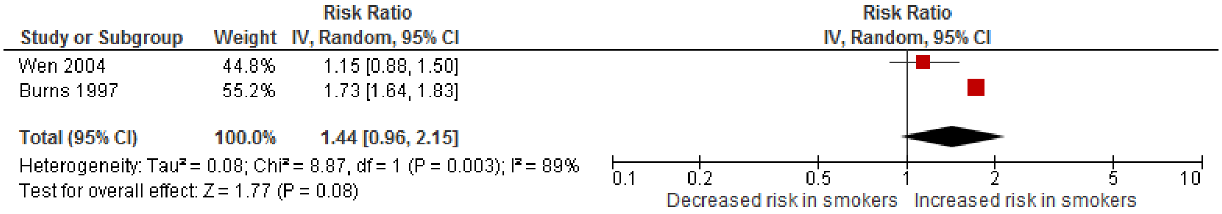

Supplementary Figure J3. Forest plots for stroke, and the relative risks associated with smoking 1, 5, 20 cigarette per day, among males aged 65 years

1 cigarette per day

Study or Subgroup	Weight	Risk Ratio IV, Random, 95% CI
Xu 2007	17.4%	1.06 [0.77, 1.46]
LaCroix 1991	12.3%	1.14 [0.59, 2.20]
Ji 2011	17.3%	1.16 [0.83, 1.61]
Pham 2007	14.0%	1.17 [0.68, 2.03]
Kondo 2011	8.3%	1.84 [0.68, 4.96]
Ehteshami-Afshar 2014	11.8%	1.92 [0.95, 3.85]
Tverdal 2011	19.0%	2.50 [2.06, 3.04]
**Total (95% CI)**	**100.0%**	**1.45 [1.00, 2.11]**

Heterogeneity: $Tau^2 = 0.18$; $Chi^2 = 31.96$, df = 6 (P < 0.0001); $I^2 = 81\%$
Test for overall effect: Z = 1.97 (P = 0.05)

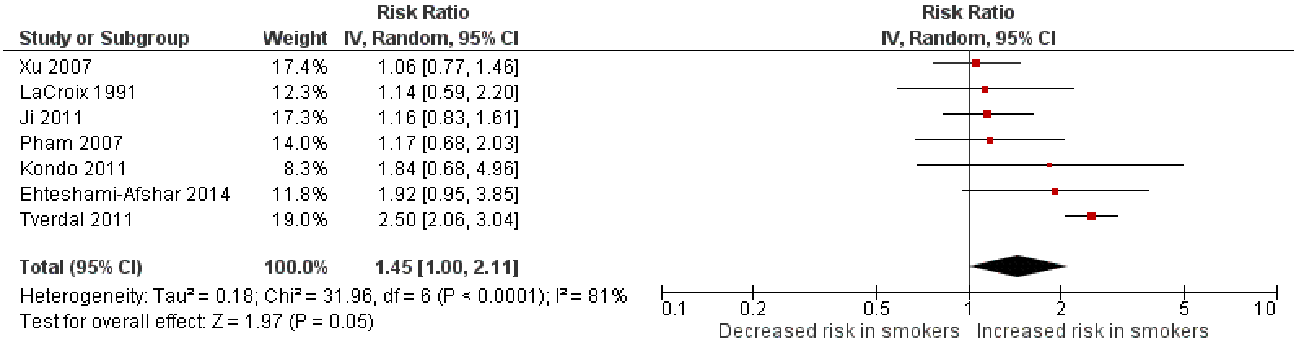

5 cigarettes per day

Study or Subgroup	Weight	Risk Ratio IV, Random, 95% CI
Xu 2007	16.5%	1.12 [0.86, 1.44]
Pham 2007	14.2%	1.20 [0.77, 1.86]
LaCroix 1991	12.9%	1.25 [0.74, 2.13]
Ji 2011	16.5%	1.30 [1.00, 1.68]
Kondo 2011	9.6%	2.05 [0.93, 4.48]
Ehteshami-Afshar 2014	12.7%	2.39 [1.39, 4.12]
Tverdal 2011	17.5%	2.59 [2.22, 3.03]
**Total (95% CI)**	**100.0%**	**1.59 [1.11, 2.26]**

Heterogeneity: $Tau^2 = 0.18$; $Chi^2 = 46.90$, df = 6 (P < 0.00001); $I^2 = 87\%$
Test for overall effect: Z = 2.56 (P = 0.01)

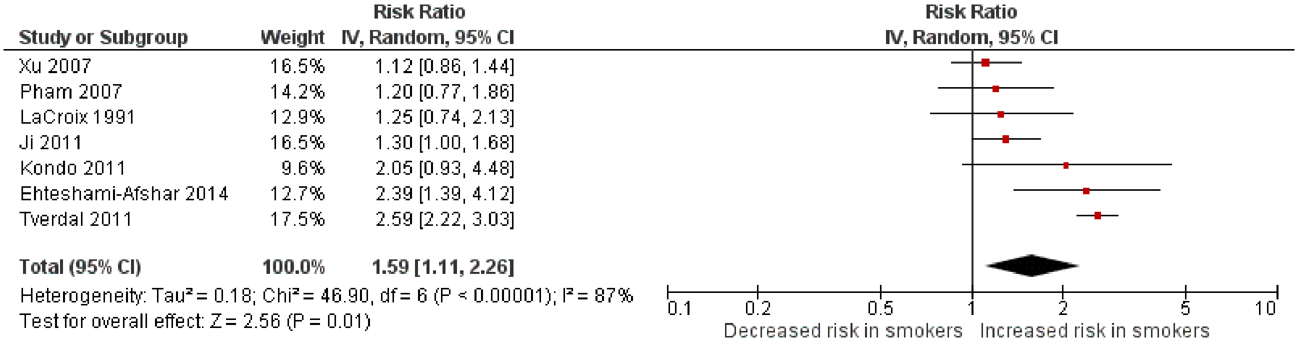

20 cigarettes per day

Study or Subgroup	Weight	Risk Ratio IV, Random, 95% CI
Pham 2007	14.6%	1.32 [1.00, 1.74]
Xu 2007	15.8%	1.35 [1.19, 1.54]
LaCroix 1991	14.7%	1.79 [1.37, 2.33]
Ji 2011	15.1%	1.96 [1.57, 2.46]
Tverdal 2011	15.8%	2.96 [2.64, 3.31]
Kondo 2011	12.2%	3.08 [1.91, 4.97]
Ehteshami-Afshar 2014	11.8%	5.50 [3.29, 9.19]
**Total (95% CI)**	**100.0%**	**2.19 [1.56, 3.09]**

Heterogeneity: $Tau^2 = 0.19$; $Chi^2 = 107.13$, df = 6 (P < 0.00001); $I^2 = 94\%$
Test for overall effect: Z = 4.49 (P < 0.00001)

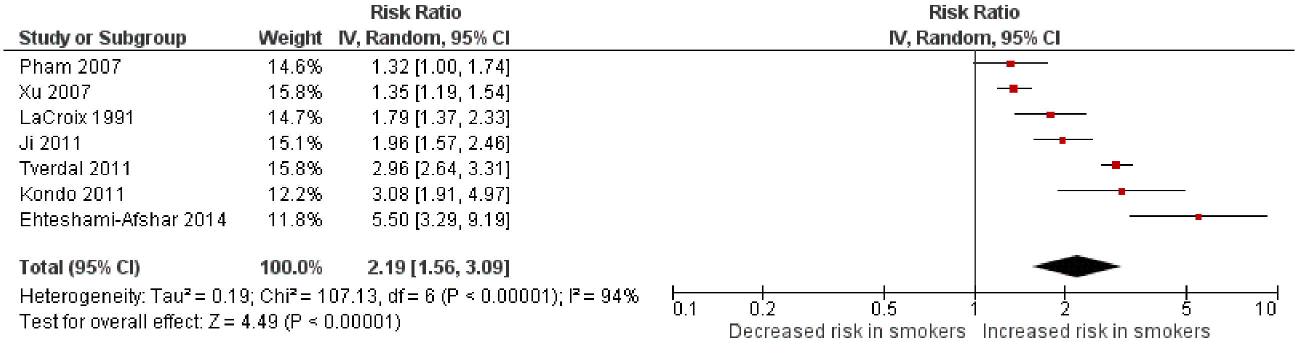

Supplementary Figure K. Forest plots for cardiovascular disease (coronary heart disease and stroke not reported separately) and the age-adjusted relative risks associated with smoking 1, 5 or 20 cigarettes/day among males

1 cigarette per day

Study or Subgroup	Weight	Risk Ratio IV, Random, 95% CI
Pope 2009	78.6%	1.61 [1.50, 1.73]
Gellert 2013	3.7%	1.68 [1.22, 2.31]
Huxley 2012	1.9%	1.71 [1.08, 2.69]
Mons 2015	15.8%	1.71 [1.46, 2.00]
**Total (95% CI)**	**100.0%**	**1.63 [1.53, 1.73]**

Heterogeneity: $Tau^2 = 0.00$; $Chi^2 = 0.58$, $df = 3$ ($P = 0.90$); $I^2 = 0\%$
Test for overall effect: $Z = 15.43$ ($P < 0.00001$)

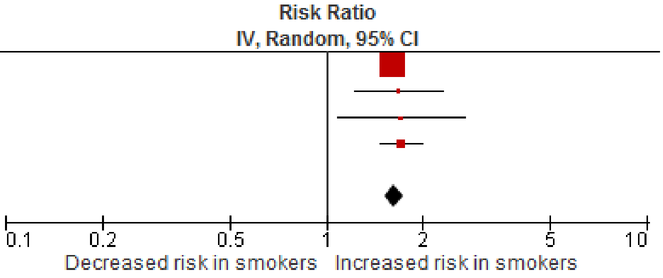

5 cigarettes per day

Study or Subgroup	Weight	Risk Ratio IV, Random, 95% CI
Pope 2009	78.3%	1.68 [1.59, 1.77]
Gellert 2013	3.9%	1.82 [1.42, 2.34]
Mons 2015	16.0%	1.83 [1.62, 2.07]
Huxley 2012	1.8%	1.84 [1.27, 2.67]
**Total (95% CI)**	**100.0%**	**1.71 [1.63, 1.80]**

Heterogeneity: $Tau^2 = 0.00$; $Chi^2 = 2.01$, $df = 3$ ($P = 0.57$); $I^2 = 0\%$
Test for overall effect: $Z = 21.24$ ($P < 0.00001$)

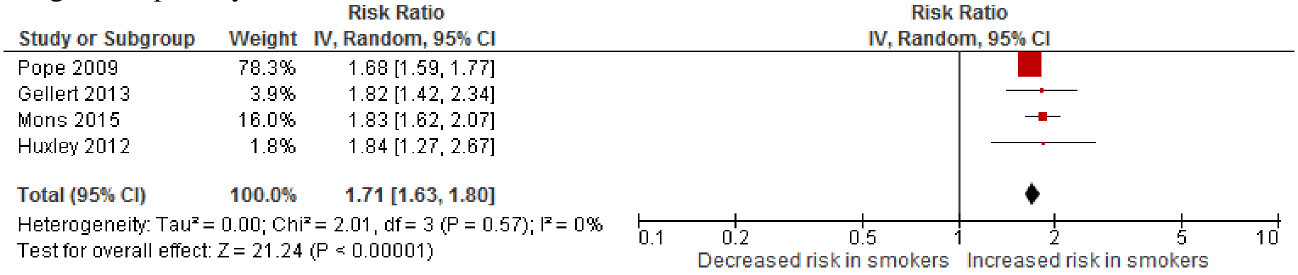

20 cigarettes per day

Study or Subgroup	Weight	Risk Ratio IV, Random, 95% CI
Pope 2009	32.2%	1.97 [1.91, 2.02]
Mons 2015	27.7%	2.36 [2.12, 2.63]
Huxley 2012	20.8%	2.46 [2.03, 2.97]
Gellert 2013	19.3%	2.48 [2.01, 3.06]
**Total (95% CI)**	**100.0%**	**2.27 [1.96, 2.62]**

Heterogeneity: $Tau^2 = 0.02$; $Chi^2 = 19.32$, $df = 3$ ($P = 0.0002$); $I^2 = 84\%$
Test for overall effect: $Z = 11.16$ ($P < 0.00001$)

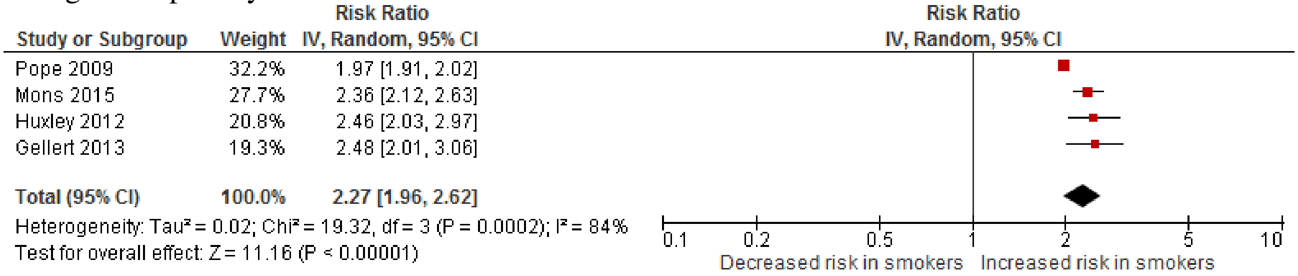

Supplementary Figure L. Forest plots for cardiovascular disease (coronary heart disease and stroke not reported separately) and the age- and sex-adjusted relative risks associated with smoking 1, 5 or 20 cigarettes/day (for studies that did not separate males and females).

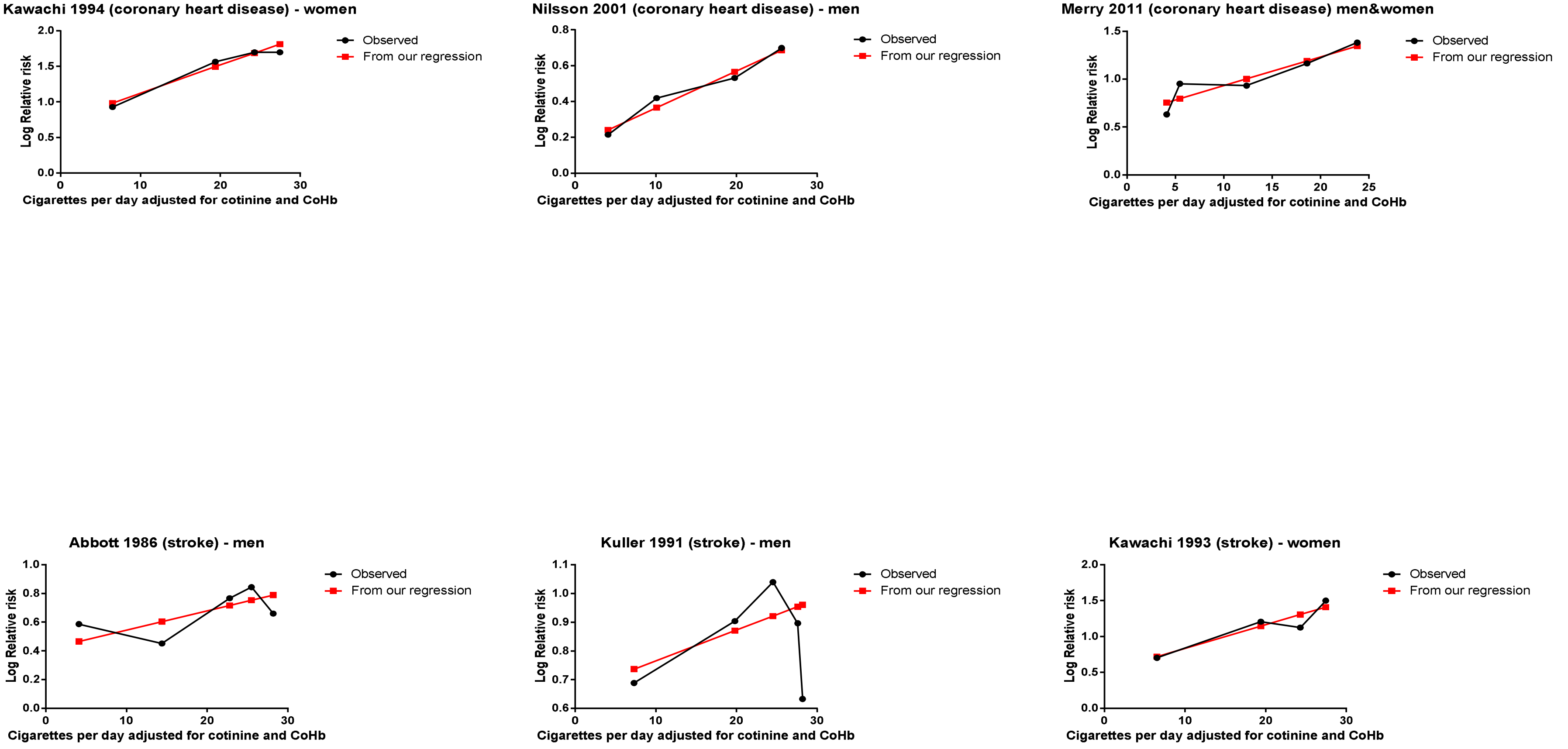

Supplementary Figure M. Examples of studies showing the extent of fit between the observed (reported) relative risks and the estimates we produced from the log-linear regressions. Focus is on best fit at 1 and 20 cigarettes per day, rather than the whole dose-response relationship.

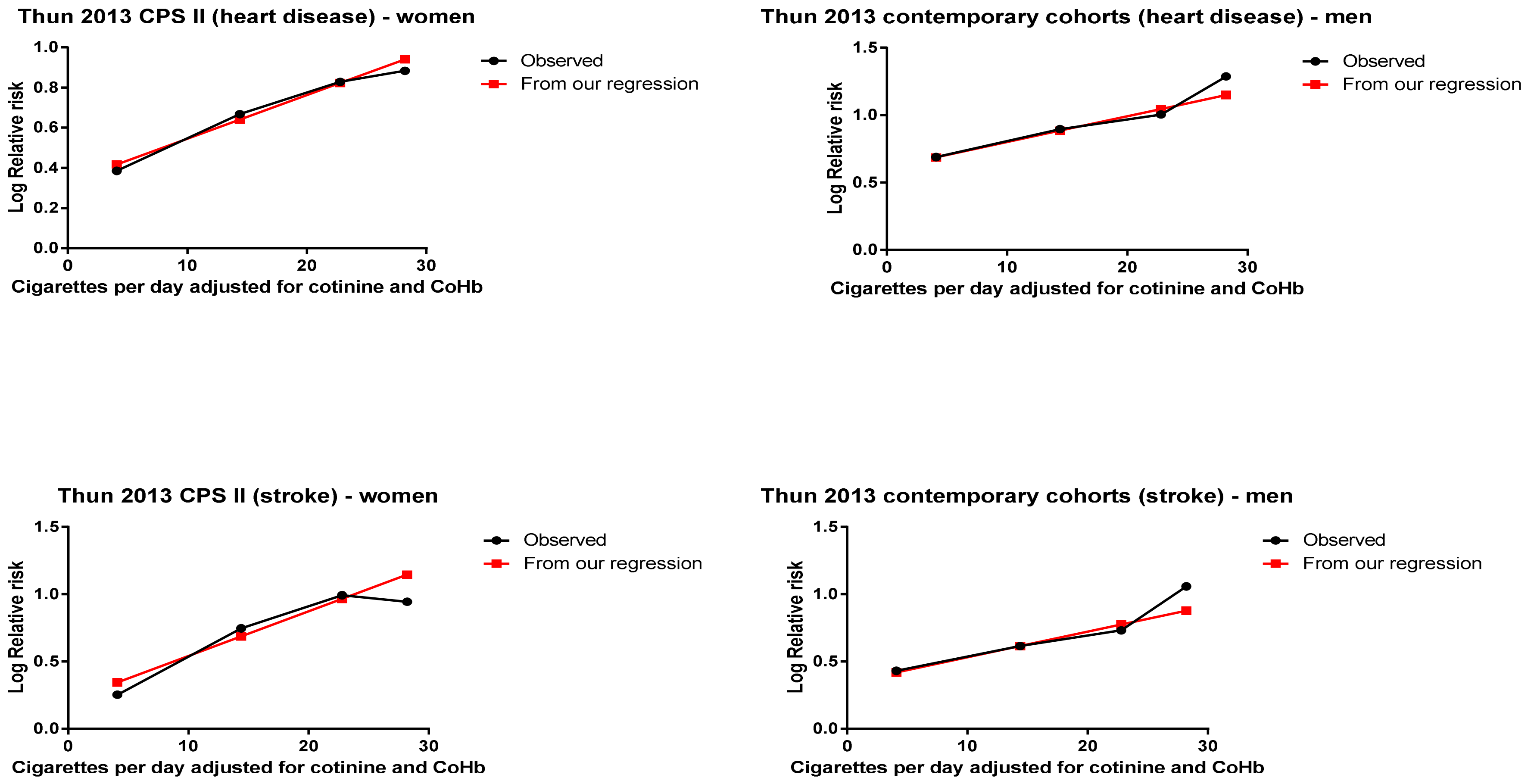

Supplementary Figure M. continued.

Supplementary Table A. Country, years of recruitment and confounders in the 55 study reports

Reference	First author, year	Country	Years of recruitment	Effect size used	Confounders adjusted for*
16	Abbott 1986	Hawaii	1956-1968	HR	Age
17	Bjartveit 2005	Norway	1972-1978	HR	Age, BP, cholesterol, triglyceride, physical activity, BMI, height
18	Burns 1997	USA	1959-1960	RR	Separate analyses by age and sex
19	Bush 1983	USA	1963	RR	Marital status, education, housing quality
20	Doll 1980	UK	1951	RR	Age
21	Doll 2004	UK	1951	RR	Age, time period
22	Ehteshami-Afshar 2014	Iran	1999-2001	HR	Age, diabetes, hypertension, duration of smoking, hypercholesterolemia, BMI, family history CVD, marital status, education
23	Freund 1993	USA	1948-1952	HR	Separate analyses by age and sex
24	Fuller 1983	UK	1967-1969	RR	Age
25	Gellert 2013	Germany	2000-2002	HR	Age, sex, education, alcohol, diabetes, BMI, BP, cholesterol, physical activity
26	Gun 2006	Australia	1980-1983	RR	Age
27	Hart 2000	UK	1972-1976	HR	Age
28	Hippisley-Cox 2013	UK	1998-2012	HR	Age, BP, cholesterol, deprivation score, ethnicity, family history
29	Hirayama 1990	Japan	1965	RR	Age
30	Honjo 2010	Japan	1980-1990	HR	Age, cohort
31	Huxley 2012	USA	1987-1989	HR	Age, sex, location, education, income, alcohol, physical activity, BP, BP-medication, diabetes, cholesterol
32	Iversen 2013	Norway	1974	HR	Age, BP, cholesterol, BMI, physical activity, passive smoking
33	Jacobs 1999	Europe, USA, Japan	1957-1964	RR	Age, country
34	Jamrozik 2011	Australia	1996	HR	Age, location, country of birth, education, marital status
35	Ji 2011	China	1974-1980	HR	Age, BMI, BP, cholesterol
36	Jonsdottir 2002	Iceland	1967-1991	HR	Age, BP, hypertension, cholesterol, triglyceride, diabetes, glucose level, BMI, angina
37	Kahn 1966	USA	1954	RR	Separate analyses by age (men only)
38	Kawachi 1993	USA	1976	HR	Age, hypertension, diabetes, hypercholesterolemia, BMI, prior use oral contraceptives, estrogen therapy, age start smoking
39	Kawachi 1994	USA	1976	HR	Age, hypertension, diabetes, hypercholesterolemia, BMI, prior use oral contraceptives, estrogen therapy, menopausal status, age start smoking
40	Kelly 2008	China	1991	HR	Age, education, alcohol, physical activity, BMI, BP, location, urbanisation, diabetes, previous heart disease
41	Khang 2008	South Korea	1994	HR	Age
42	Kondo 2011	Japan	2000-2008	HR	Age, BP, cholesterol, glucose level
43	Kono 1985	Japan	1965	HR	Age
44	Kuller 1991	USA	1972	HR	Age
45	LaCroix 1991	USA	1981-1983	HR	Age, location
46	Lam 2002	China	1987	HR	Age, BP, BMI, cholesterol, triglyceride, alcohol, physical activity
47	Lam 2007	Hong Kong	1998-2000	HR	Age, BMI, education, alcohol, physical activity, active chronic disease, hypertension, diabetes, hypercholesterolemia, COPD/asthma, regular medication use, prior hospital admission, expenditure, recent unintentional weight loss, self-rated health, functional disability, depression symptoms
48	Lawlor 2008	South Korea	1992	HR	Age, height, BP, BMI, cholesterol, hyperglycemia, alcohol, physical activity, location

49	Liaw 1998	Taiwan	1982-1986	HR	Age, sex, BP
50	Merry 2011	Netherlands	1987-1997	HR	Age, sex, cohort, alcohol, diabetes, education, family history heart disease, cholesterol, BP, BMI
51	Molshatzki 2013	Israel	1963	HR	Age, BP, diabetes, BMI, socioceconomic status, hardship score
52	Mons 2015	Europe, USA, Russia	1979-2008	HR	Age, sex, education, alcohol, BMI, physical activity#
53	Nilsson 2001	Sweden	1963	HR	Age, location
54	Pham 2007	Japan	1986-1989	HR	Age, BMI, alcohol, vegetable consumption, diabetes, employment status, study area
55	Pirie 2013	UK	1996-2001	HR	Age, location, BMI, deprivation score, alcohol, physical activity, height, oral contraceptive use, menopausal status, menopausal hormone therapy
56	Pooling Project 1978	USA	<1970	RR	Separate analyses by age and sex
57	Pope 2009	USA	1982	HR	Age, sex, ethnicity, education, marital status, BMI, alcohol, diet, occupational exposures
58	Prescott 1998	Denmark	~1980	HR	Age, study cohort, BP, cholesterol, triglyceride, BMI, education, alcohol, physical activity, height
59	Ragland 1988	USA	1960-1961	HR	Separate analyses by age and sex
60	Shaper 2003	UK	1978-1989	HR	Age, BMI, BP, social class, cholesterol, alcohol, BP-therapy use
61	Shapiro 1969	USA	1961	RR	Age
62	Thun 2013	USA	1959-1996	HR	Age, ethnicity, education, cohort
63	Tverdal 2011	Norway	1974-1978	HR	Age, BP, cholesterol, triglyceride, physical activity, BMI, height, disability pension, sickness leave, family history heart disease
64	Watt 1995	UK	1964-1976	HR	Age
65	Weir 1970	USA	1954-1957	RR	Separate analyses by age and sex
66	Wen 2004	Taiwan	1982-1992	HR	Age
67	Woodward 1999	UK	1984-1987	HR	Age, cholesterol, BP
68	Woodward 2005	Asia-Pacific	1961-1998	HR	Age, BP
69	Xu 2007	China	1996-2000	HR	Age, BMI, education, history of cancer, chronic bronchitis, hypertension, alcohol
70	Zhang 2011	Germany	1984-1990	HR	Age, cohort, alcohol, hypertension, cholesterol, physical activity, diabetes

*where sex has not been indicated, it means that separate analyses were provided for males and females
#the authors stated that additional adjustment for diabetes, BP, and cholesterol did not materially change the results.

BP: blood pressure
BMI: body mass index
RR: relative risk (from comparison of incidence)
HR: hazard ratio from Cox proportional hazards regression
Location: place of residence

The studies in reference numbers 16, 44, 51, 59, 65, 66, and 68 might have included former smokers in the group of non-smokers used as the reference group for the relative risks/hazard ratios.

Supplementary Table B. Individual cohort studies showing the observed age-adjusted relative risks for developing coronary heart disease or stroke in smokers who consume up to around 5 cigarettes per day (each relative risk has the reference category of 1.0 for never-smokers), and for around 20 per day. The numbers in brackets are the relative risks estimated from our regression modelling (used in the meta-analyses).

		Lowest smoking category (estimated for 1 or 5 per day)	Upper smoking category (estimated for 20 per day)	Proportion of excess risk for light compared to heavy smoking (median=56%; 49% CHD, 62% stroke)*
Hirayama 1990[29]	Men	1-4 per day	20-24 per day	
	Heart disease	1.68	1.90	75
	Stroke	1.50	0.99	>100%
	Women			
	Heart disease	1.61	2.39	44%
	Stroke	1.20	1.32	62%
Rosengren 1992[71]	Men	1-4 per day	15-24 per day	
	Heart disease	2.8	3.1	86%
Kawachi 1994[39]	Women	1-4 per day	15-24 per day	
	Heart disease	1.94 (2.15/2.52)	4.22 (4.56)	29%
Jacobs 1999[33]	Men	1-4 per day	20-29 per day	
	Heart disease	1.12 (0.97/1.05)	1.45 (1.41)	27%
	Stroke	0.88 (1.04/1.06)	1.10 (1.13)	-20%
Nilsson 2001[53]	Heart disease	1-7 per day	16-25 per day	
	Men	1.24 (1.19/1.30)	2.24 (1.82)	19%
	Women	1.47 (1.36/1.50)	1.70 (2.19)	67%
Prescott 2002[72]#	Heart disease	3-5 per day	15-24 per day	
	Men	1.03 (1.58/1.62)	1.61 (1.76)	5%
	Women	2.14 (2.33/2.50)	3.15 (3.28)	53%
Bjartveit 2005[17]	Heart disease	1-4 per day	20-24 per day	
	Men	2.74 (2.48/2.69)	3.75 (3.63)	63%
	Women	2.94 (3.15/3.44)	4.25 (4.75)	60%
Pope 2009[57]	Men+women	1-3 per day	18-22 per day	
	Heart disease	1.63 (1.66/1.72)	1.98 (1.93)	64%
	CVD	1.64 (1.61/1.68)	2.02 (1.97)	63%
Tverdal 2011[63]	Stroke	1-4 per day	15+ per day	
	Men	2.16	2.25	93%
Merry 2011[50]	Men+women	1-5 per day	16-20 per day	
	Heart disease	1.88 (1.94/2.19)	3.20 (3.42)	40%
Pirie 2013[55]	Women	5 per day	20 per day	
	Heart disease	~2.1 (2.38/2.79)	~5.2 (5.08)	26%
	Stroke	~1.6 (1.84/2.11)	~3.5 (3.51)	24%

CVD: all cardiovascular disease
The observed relative risks are based on reported cigarette consumption which has not allowed for extent of inhalation, i.e. CoHb and cotinine (as we have done in our meta-analyses, see Methods section).

*The excess relative risk for light smoking expressed as a proportion of that for heavy smoking (e.g. for Nilsson 2001, it is (1.24-1)/(2.24-1)=0.19 (19%).

#Overlaps with Prescott 1998[58], but the 1998 report was used in the meta-analyses because it contained more study cohorts (hence more participants). The modelled estimates in the table above use the 2002 data.

The death rate per 100,000 was 718 for those smoking 1-4 cigarettes/day but lower for 20-24 per day (472 per 100,000)

Rosengren 1992 and Tverdal 2011 (stroke; men) do not appear in the meta-analyses because when all smoking categories were analysed the regression (spuriously) produced a negative slope between consumption and risk, which is implausible given the established causal dose-response relationship. They are shown in the above table for interest.
